# Machine learning models outperform deep learning models, provide interpretation and facilitate feature selection for soybean trait prediction

**DOI:** 10.1186/s12870-022-03559-z

**Published:** 2022-04-08

**Authors:** Mitchell Gill, Robyn Anderson, Haifei Hu, Mohammed Bennamoun, Jakob Petereit, Babu Valliyodan, Henry T. Nguyen, Jacqueline Batley, Philipp E. Bayer, David Edwards

**Affiliations:** 1 School of Biological Sciences and Institute of Agriculture, University of Western Australia, Perth, WA Australia; 2 Department of Computer Science and Software Engineering, The University of Western Australia, Perth, WA Australia; 3 Division of Plant Sciences and National Center for Soybean Biotechnology, University of Missouri, Columbia, MO 65211 USA; 4 Department of Agriculture and Environmental Sciences, Lincoln University, Jefferson City, MO 65101 USA

**Keywords:** Machine learning, XGBoost, Interpretable models, Feature selection, Genomic selection, Soybean

## Abstract

Recent growth in crop genomic and trait data have opened opportunities for the application of novel approaches to accelerate crop improvement. Machine learning and deep learning are at the forefront of prediction-based data analysis. However, few approaches for genotype to phenotype prediction compare machine learning with deep learning and further interpret the models that support the predictions. This study uses genome wide molecular markers and traits across 1110 soybean individuals to develop accurate prediction models. For 13/14 sets of predictions, XGBoost or random forest outperformed deep learning models in prediction performance. Top ranked SNPs by F-score were identified from XGBoost, and with further investigation found overlap with significantly associated loci identified from GWAS and previous literature. Feature importance rankings were used to reduce marker input by up to 90%, and subsequent models maintained or improved their prediction performance. These findings support interpretable machine learning as an approach for genomic based prediction of traits in soybean and other crops.

**Supplementary Information:**

The online version contains supplementary material available at 10.1186/s12870-022-03559-z.

## Introduction

Soybean (*Glycine max*) has a variety of uses including human consumption, livestock and aquaculture feed, and biofuel production [1, 2]. The demand for soybean is expected to increase [3], whilst climate change is expected to decrease overall crop productivity, threatening global food security [4]. The production of large quantities of genomic data in the last 10-15 years has supported the development of genomics-based approaches for crop improvement that can address these challenges [5]. Genomic Selection (GS) has been applied to associate Single Nucleotide Polymorphisms (SNPs) with breeding values to accelerate crop improvement. GS has the potential to reduce breeding cycle length [6] and accelerate genetic gains in crops by improving breeding selection [7], supported by methods such as speed breeding [8].

Studies have shown that using non-linear prediction algorithms such as Machine Learning (ML) can improve prediction accuracy in GS [9–11]. The application of ML in crop breeding provides advantages such as the use of more complex data, along with potentially providing solutions to problems such as epistasic effects and genomic imprinting [12]. A relatively new subcategory of ML, Deep Learning (DL), has provided promising results in a range of fields and disciplines using interconnected neural networks such as Convolutional Neural Networks (CNN) and Deep Neural Networks (DNN). The advantage of DL is that when optimised appropriately, it can identify complex multidimensional patterns in large data sets [13]. CNNs specifically have already demonstrated this for GS through its application in selecting high value phenotypes from genomic data [6].

The underlying mechanisms of genotype to phenotype predictions remain unclear. Adding associated SNPs from Genome Wide Association Studies (GWAS) into linear prediction models has shown varying results [14, 15]. For non-linear models, tools are being developed to increase the accuracy of predictions in ML and DL using GWAS data [16]. The changes in accuracy using GWAS related inputs suggests that prediction models can, for certain traits, place importance on these SNPs for building the model. Identification of these loci provides the opportunity to guide the reduction of input data through feature selection in further models.

For genotype to phenotype predictions, it is appropriate to test a range of ML/DL algorithms as each algorithm has its own underlying assumptions and biases, and no algorithm provides the best performance for all traits [17]. Studies in this area often compare DL models to linear models and with older ML models such as random forest [18], but often forgo the inclusion of one of the most recent ML models, XGBoost. This omission is of concern due to XGBoost’s accurate prediction in other disciplines [19–21], and XGBoost has shown to outperform DL in some tabular data problems [22].

In this study we build robust prediction models for seven agronomic traits in soybean and compare the suitability of prediction models for use in crop breeding. Our results suggest that XGBoost has an affinity for genotype to phenotype prediction and should be considered in future model development. The trait associated regions identified by XGBoost overlap with regions of significantly associated loci from GWAS, and XGBoost can independently identify these regions. Furthermore, reducing the input data to a targeted selection of SNPs based upon initial regions of importance to XGBoost tree building can provide equal or better results using far fewer SNPs.

## Methods

### SNP discovery & phenotype data

A total of 1110 diverse soybean accessions were selected from the USDA Soybean Germplasm Collection for SNP calling [23]. The sequence metadata for all 1110 soybean accessions are summarized in Table S2. Clean reads were mapped to the pangenome using BWA-MEM [24] v0.7.17 with default settings and duplicates removed by Picard tools (http://broadinstitute.github.io/picard/). Reads were realigned using GATK [25] v3.8-1-0 RealignerTargetCreator and IndelRealigner, followed by variant calling using GATK HaplotypeCaller. The resulting SNPs were filtered following the SNP filtering methodology described in Marsh et al [26], (QD < 2.0 || MQ < 40.0 || FS > 60.0 || QUAL < 60.0 || MQrankSum < − 12.5 || ReadPosRankSum < − 8.0) to remove low-quality SNPs. High-confidence SNPs were identified by removing SNPs with minor allele frequency (MAF) < 0.05 and missing genotype rate < 10% using VCFtools [27]. Phenotype data for flower colour, seed coat colour, pod colour, pubescence density, seed oil content, seed protein content and seed weight were downloaded from the USDA-GRIN database (https://npgsweb.ars-grin.gov/) for the accessions that had available observation data. The range of phenotype data is summarised in Table S3.

### Genome wide association studies

The R package rMVP v0.99.15 [28] was used to conduct GWAS, with the FarmCPU statistical technique. FarmCPU allows for the population structure to be controlled by using the first three principal components (PCs) from an automatic principal component analysis (PCA) based on the marker data [29], whilst the significance threshold was defined as 0.05/marker size. GWAS was run with rMVP using the following settings, nPC.FarmCPU = 3, priority = “memory”, vc.method = “BRENT”, maxLoop = 10, method.bin = “EMMA”, threshold = 0.05.

### Data pre-processing for machine learning model building

Vcftools [27] vcf-to-tab was used to remove vcf preamble. A python script was used to reformat this file into a structured csv. Soybean lines with over 1% missing data were excluded.

SNPs were reduced by 95% by extracting 1 in 20 sequential SNPs, as our initial total of approximately 5 million SNPs was not compatible with GPU memory requirements. For each trait, 20% of samples were randomly excluded to create a holdout set by using the packages pandas and numpy within a python script before model building. This holdout data was later used for model validation.

For models with reduced feature input, SNPs for genomic regions based on XGBoost feature importance were extracted using a custom python script. Holdout sets were produced in the same manner as for the complete dataset.

### Model building and feature importance

A virtual sandbox environment was built on GPU servers using singularity with a tensorflow docker image (version ‘tensorflow:20.03-tf2-py3’). Jupyter notebooks were connected to the server, and are available at https://github.com/mitchgill16/Soybean_Trait_Prediction. The python package Scikit learn v0.21 [30] was used to perform a variety of ML relevant tasks.

For multiclass classification problems, XGBClassifier was initiated with objective = ‘multiclass’ and num_classes set to the number of trait classes. For other regression and classification problems, XGRegressor and XGBClassifier were initiated with default settings, all of which were loaded in with the XGBoost package v1.1.1. XGBoost model parameters were optimised using the BayesSearchCV object from scikit optimisation package v0.8.1. The setting space was follows: learning_rate(0.01 - 1.0), min_child_weight(0 - 10), max_depth(0 - 50), max_delta_step(0 - 20), subsample(0.01 - 1.0), colsample_bytree(0.01 - 1.0), colsample_bylevel(0.01 - 1.0), reg_lambda(1e^9 - 1000), reg_alpha(1e^9 - 1.0), gamma(1e-9 – 0.5), min_child_weight(0 - 5), n_estimators(50 – 200) and scale_pos_weight(1e^6 - 500). The optimum XGBoost parameters were then used to fit the XGBoost models using stratified k-fold cross validation for classification tasks, and standard k-fold cross validation (k = 10) from the scikit learn model selection package. The best performing model from cross validation was saved using the inbuilt pickle python package (protocol version 5) and used for prediction on the holdout dataset.

The functions ‘get_booster’ and ‘get_scores’ was used to generate dictionaries of importance scores for each feature used by the XGBoost model. The top 20 scores were retrieved and ranked. Original SNP names were retrieved from a stored list of headers, and allele values were retrieved by a custom inverse one hot encoding function.

Random forest objects for classification (RandomForestClassifier), and continuous (RandomForestRegressor) traits were loaded from the scikit learn ensemble python package. Both the random forest classifier object and random forest regressor object were initialised with n = estimators = 100, max_features = “sqrt” and the random state set to a random integer between 0 and 5000. To ensure an optimised and fitted model could be selected for use on the holdout dataset, the random forest objects were fitted using stratified k-fold cross validation for classification tasks, and standard k-fold cross validation (k = 10) from the scikit learn model selection package. The best performing model from all folds during cross validation was selected and subsequently saved using the inbuilt pickle python package and used for prediction on the holdout dataset.

The Keras 2.4.3 interface (https://github.com/keras-team/keras) for the Tensorflow v2.1.0 [31] python library was used to build sequential DL models. The CNN architecture was initially adapted from the successful models in the GMStool [16] as a baseline for further adjustment, whilst the DNN architecture involved adapting elements from both the GMStool paper and a successful DNN architecture for prediction of yield [32]. The final hyperparameter and architecture choices were a mixture of trial and error, grid searching and adaption from the aforementioned papers.

The CNN models used three 1D convolution layers using Rectified Linear Unit (ReLU) activation, with a 20% dropout between layer one and two, and a 10% dropout between layer two and three. The convolution layers had 12, 10 and 8 filters and a kernel size of 14, 10 and 8 respectively. The convolution layers were followed by a 1D max pooling layer of size 2 and batch normalisation before being flattened and fed into three dense layers. These dense layers had 48, 32 and 16 nodes, each with ReLU activation and were followed by a batch normalisation layer.

The DNN models for this study were a fully connected feed forward multilayer perceptron network consisting of five dense layers with ReLU activation functions, a dropout layer of 3, 2 and 1% after the first three layers respectively, and a batch normalisation layer after the final dense layer. The layers consisted of 200, 100, 64, 32 and 16 nodes each. For both CNN’s and DNN’s, the output layer had one node with linear activation for continuous traits, 1 node with sigmoid activation for binary categorical traits and x nodes with softmax activation for multiclass traits, where x = the number of possible classes. The optimiser used was Adamax with a learning rate of 0.003. The batch size was 1/50th of the total amount of samples to ensure memory could be managed when running through the neural network.

## Results

**Fig. 1:**
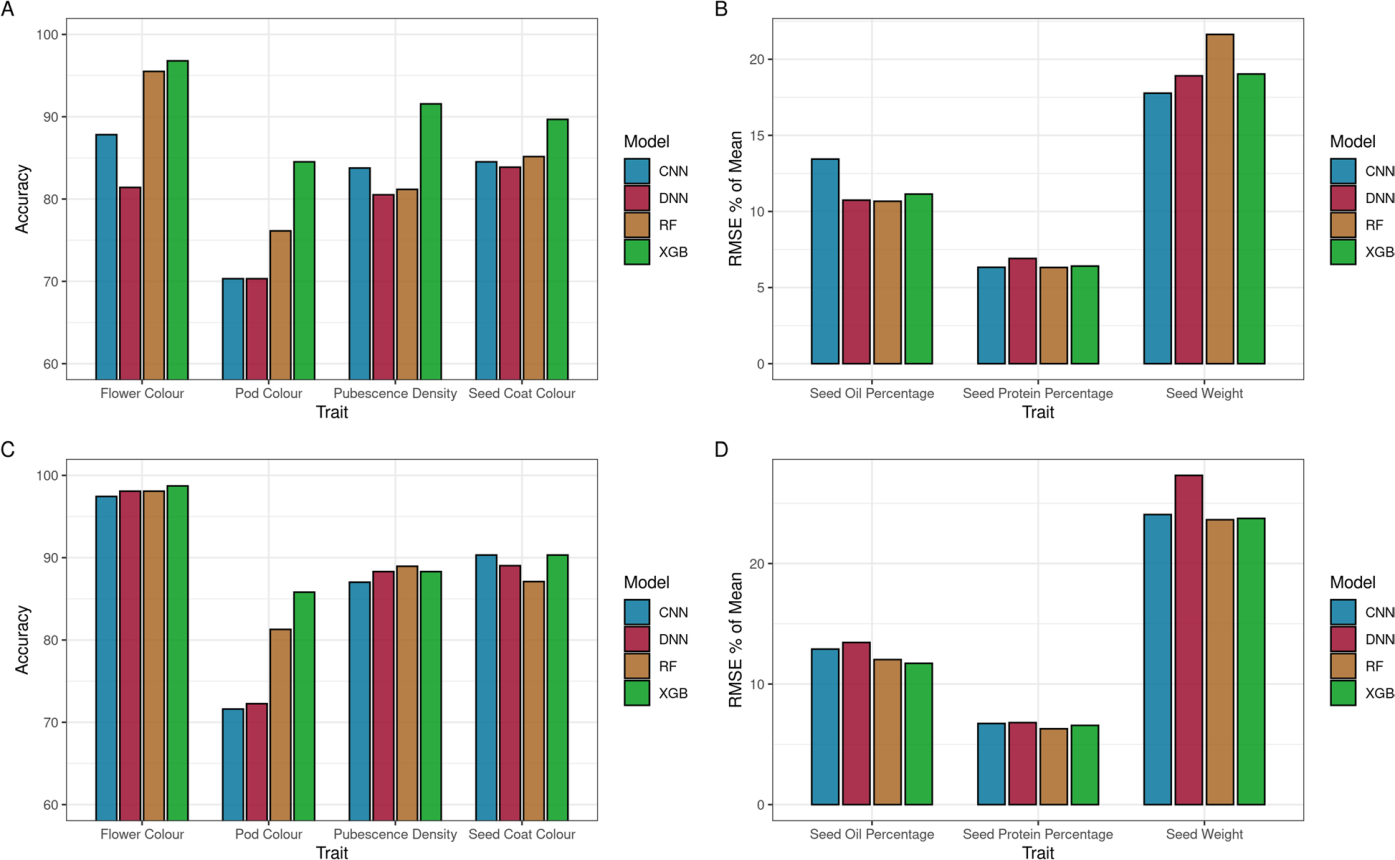
Model Prediction Performance Across Soybean Traits. **A** Accuracy for flower colour, pod colour, pubescence density and seed coat colour for models trained on SNP input data uniformly distributed across the soybean genome. **B** Root mean square error as a percentage of mean trait value for seed oil as a percentage of total seed weight, seed protein as a percentage of total seed weight and total seed weight. Models were trained on SNP input data uniformly distributed across the soybean genome. **C** Accuracy for flower colour, pod colour, pubescence density and seed coat colour for models trained on reduced SNP input data set. **D** Root mean square error as a percentage of mean trait value for seed oil as a percentage of total seed weight, seed protein as a percentage of total seed weight and total seed weight. Models were trained on a reduced SNP input data set

XGBoost, random forest, CNN and DNN models were trained and evaluated on SNP input data uniformly distributed across the genome to predict each of the following traits: flower colour, pod colour, pubescence density, seed coat colour, seed oil content, seed protein content and seed weight (Table 1, Fig. 1). Categorical traits were evaluated with classification accuracy, whereas continuous traits used Root Mean Squared Error (RMSE) as a percentage of trait mean to evaluate models and allow a comparison of prediction error across traits and models. XGBoost models outperformed other models for all categorical traits, however for the continuous traits of seed oil and seed protein percentage, random forest was the best performer, and for seed weight the CNN was most accurate. In comparison to the DL architectures (CNN & DNN), XGBoost was on average 10.32% more accurate across classification predictions. XGBoost’s performance in comparison to DL prediction error for traits requiring regression analysis was negligible, with an average of 0.16% reduction in error for regression traits when compared to DL prediction error. The best performing XGBoost model on each continuous trait was outperformed by the trained DNN for seed oil prediction, the trained CNN for seed protein prediction and both the CNN and DNN models for seed weight prediction. Like XGBoost, random forest on average performed better than DL models for classification traits, with a 4.17% increase in accuracy when compared to DL accuracy across classification prediction. Random forest models also had the lowest error for both seed oil and seed protein prediction.

**Table 1:**
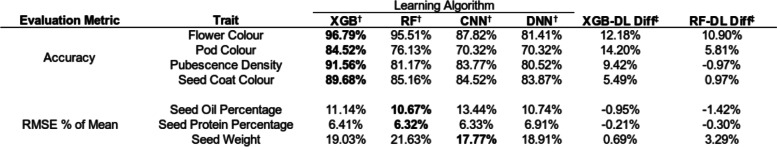
Evaluation and comparison of prediction models on whole genome SNP input data

^†^XGB = XGBoost, RF = Random Forest, CNN = Convolutional Neural Network, DNN = Deep Neural Network

^‡^XGB-DL Diff = Difference in performance between XGBoost & deep learning architectures, RF-DL Diff = Difference in performance between random forest and deep learning architectures

To compare the results and assess the potential to reduce input data we interpreted the XGBoost models and found SNPs that were important for prediction. For each trait, the XGBoost input SNPs were ranked by their F-score, measured in gain, which is a measure of the relative contribution of each SNP. For flower colour, seed coat colour, pubescence density and seed weight, the 20 highest ranking SNPs by XGBoost F-score included subsets of at least 3 SNPs within close proximity. Each SNP in a subset was separated by a maximum of 100kbp from their nearest neighbouring SNP within a given subset. The genetic regions that these subsets of SNPs spanned were defined as regions of importance (ROI), of which 6 in total were identified across 4 traits, and are summarised in Table 2. To investigate the ROIs further we performed a GWAS with all available SNPs to determine whether any of the ROI overlapped with loci identified from GWAS. GWAS identified one major loci for each of flower colour, seed coat colour and pod colour (Table 3) (Fig. S1). A flower colour ROI and seed coat colour ROI overlapped with significant GWAS loci for their respective trait, whereas the seed weight ROI did not overlap with any significant GWAS loci. There were no significant regions identified for pubescence density using GWAS, however a region identified from our XGBoost models overlaps with a previously identified locus on chromosome 12 for pubescence density [33]. In summary, the majority of the significant GWAS loci identified in this study had an overlapping ROI whilst three out of six ROI identified had an overlapping significant GWAS loci from this study and a previous study.

**Table 2:**
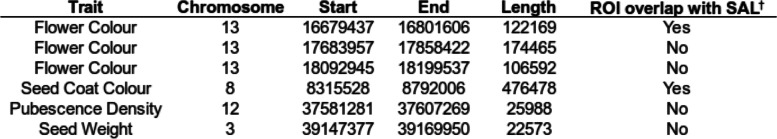
Regions of Importance (ROI) from XGBoost

^†^SAL = Significantly Associated Loci identified from GWAS

**Table 3:**
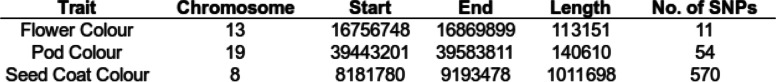
Significantly Associated Loci (SAL) from GWAS

We selected a subset of targeted SNPs for each trait based on the XGBoost interpretation (Figs. S2-S5, Table S1). Input data was reduced, retaining between 27 and 4% depending on the trait being predicted. Subsequent models for predicting discrete traits showed an increase in accuracy across all classification predictions, ranging from 2.42 to 7.70% (Table 4). However, models for continuous traits showed mixed results (Table 4). Seed protein prediction showed a negligible increase in average error when compared to the original models. Seed oil prediction had a minor increase in average error across models, whilst seed weight prediction had a larger increase in average error of 5.35% compared to the original models.

**Table 4:**
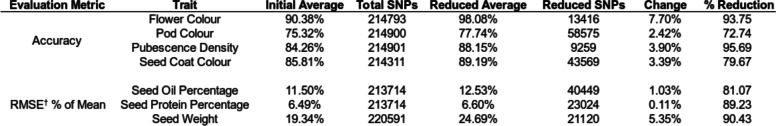
Model performance from whole genome SNP input data compared to Reduced Input SNP Data

^†^RMSE = Root Mean Square Error

When using the reduced SNP input data to evaluate the ML and DL architectures (Table 5), XGBoost performed well for classification traits as it had the highest accuracy for flower colour, pod colour and seed coat colour, while for regression traits, XGBoost had the lowest error for seed oil prediction. In comparison to the DL models, XGBoost was on average 4.03% more accurate for classification traits when using reduced SNP input data. For regression traits, XGBoost showed a 1.20% lower prediction error when compared to the average DL prediction error, and outperformed both the CNN and DNN model for each trait. Like XGBoost, random forest on average performed better than DL models for classification traits using reduced SNP input data, with a 2.10% increase in accuracy when compared to DL accuracy across classification traits. Using reduced SNP input data, random forest was the best performing model for seed oil and seed weight prediction. Random forest had a reduction in error of 1.23% when compared to DL error and outperforms both the CNN and DNN model for each of three regression traits.

**Table 5:**
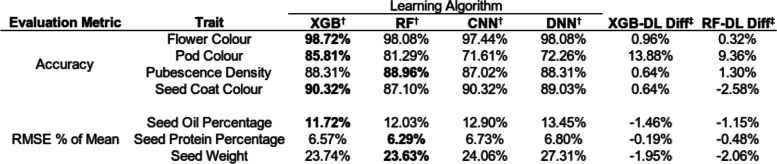
Evaluation and comparison of prediction models with reduced input data

^†^XGB = XGBoost, RF = Random Forest, CNN = Convolutional Neural Network, DNN = Deep Neural Network

^‡^XGB-DL Diff = Difference in performance between XGBoost & deep learning architectures, RF-DL Diff = Difference in performance between random forest and deep learning architectures

Across all traits with the complete SNP data, XGBoost was the best performing model for 4/7 traits, random forest for 2/7 traits, and a CNN for 1/7 traits. With reduced SNP data, XGBoost was the best performing model for 4/7 traits, and random forest for 3/7 traits. For both datasets XGBoost was the best predictor of flower colour, pod colour and seed colour whilst random forest was the best predictor of seed protein content. Overall, XGBoost was the best performer 8 of 14 times, and remained robust after feature reduction.

## Discussion

### Machine learning performance

Here we demonstrate the performance of XGBoost and random forest for genotype to phenotype predictions in comparison to widely used DL architectures. From the 14 sets of models, DL outperformed the ML models on only one occasion. The models in this study were all non-linear, which has the advantage of being able to include non-additive variances [34, 35]. Our results suggest that XGBoost and random forest models are better able to account for non-additive effects in genotype to phenotype prediction than DL architectures. The study emphasises the need to test a variety of models and provides an evaluation of the XGBoost algorithm in this research space.

Building and training effective DL models requires abundant high-quality training data [36], and for this study there was access to over 5.5 million SNPs for between 700 and 1000 individuals per trait. Khaki and Wang [32] produced successful yield prediction models in maize using over 2000 individuals, and Jeong et al. [16], trained CNNs on soybean with 1928 individuals. However it is to be noted that Jeong et al. [16], also trained successful CNN’s for rice genomic prediction on 413 individuals which suggests that sample size might not be the sole reason that a deep learning model underperforms. Whilst model optimisation and training were done according to best practices, it is possible that ML algorithms were better able to train on this smaller dataset than DL algorithms. Recent research supports this notion by concluding that random forest was able to better predict from small, tabulated datasets than DL, whilst DL was able to better predict from larger tabulated datasets [37]. Our work remains consistent with this finding and suggests that XGBoost has the ability to predict effectively from relatively small, tabulated datasets. Another consideration is recent evidence to suggest that DL models do not estimate complex marker effects, but rather use the genetic relatedness between markers to make predictions. This may partially explain the underperformance of DL in crop genomic prediction problems [38].

The importance of marker density for prediction varies between datasets, with some evidence demonstrating increasing SNP density can increase accuracy and is positively correlated with heritability [39], whereas other evidence suggests that marker density is less important, and that heritability of a trait is more important [40]. A study in cattle found that highly ranked subsets of 400-3000 SNPs provided better results than evenly spaced SNPs across the genome [41], similar to the results of our study. The ability to reduce the number of SNPs whilst retaining a high prediction performance increases the feasibility of genotyping for genomic selection with tools such as SNP arrays.

### Explainability and interpretability for genotype to phenotype prediction

XGBoost’s inbuilt methods were used to interpret the model and guide the selection of dense areas of SNPs for further model building. Using model interpretability to guide feature selection was effective, however a potential limitation of the study is that for some traits in the initial genome-wide SNP input data models, XGBoost was not the best performing model. As an alternative, model-agnostic local explanation methods such as SHAP [42] could be used on the best performing models to rank feature importance. Other methods that could be used to reduce input include QTL-based genomic assisted prediction [43], using GWAS associated SNPs [44] or using a selection of significant and non-significant SNPs from GWAS to train genomic prediction models [16].

One concern with explaining models that were built without the intention of interpretability is that it can lead to issues of validation [45], and explanations that are misleading [46]. Interpretable models such as XGBoost enable the identification of features in the underlying architecture, and support retraining to improve model classification. In addition, interpretable models allow the identification of genetic markers, and hence a genomic location for traits, providing biological context [32]. Further model building through reduction of the input space has demonstrated the ability to improve the performance across ML and DL models for trait prediction [17, 32, 47].

### Comparison and similarity of XGBoost feature importance and significantly associated regions identified through GWAS

The loci identified from GWAS for flower colour on chromosome 13, seed coat colour on chromosome 8 and pod colour on chromosome 19 are supported by previous research [48, 49]. In addition, the associated loci for flower and seed coat colour overlapped with the regions of importance identified by XGBoost. Despite being different methods, there is evidence that interpretable ML can use correlation between features and outcomes to extract associations [50], which is similar to how GWAS tests for marginal association between a target trait and SNP [51]. Our XGBoost models add evidence to this idea, as they learn which inputs to use in decisions by lowering the cumulative residual error [52], and identified loci associated with flower and seed coat colour without manual labelling to inform the model that there was a significant locus present.

This study demonstrated that XGBoost and random forest models have the ability to outperform DL architectures for genotype to phenotype prediction problems. XGBoost models and GWAS identified overlapping genomic regions for two traits. For other traits, XGBoost identified genomic regions hosting multiple SNPs that may help define new associated regions. Finally, this study used the feature importance results as a guide to reduce the number of SNPs required. These results demonstrate the feasibility of feature reduction for genotype to phenotype predictions and showcase the importance of an appropriate representation of input.

## Supplementary Information

**Additional file 1: Supplementary Figure 1.** *P*-value of each SNPs association for a) flower colour b) seed coat colour c) pod colour in the soybean VCF. SNPs coloured red have been determined as significantly associated for the given trait as they have a *p*-value less than the -log10(8) significance threshold for this GWAS. **Supplementary Figure 2.** Graphs ranking the top 20 most input SNPs by gain as identified by XGBoost models for trait predictions for traits with regions of importance identified from XGBoost. Blue bars are region of importance, whereas other colours represent collections of important SNPs on the same chromosome. Black bars represent left over SNPs with no relation to other SNPs in the ranking. SNP rankings for genome wide SNP input for A) flower colour B) seed coat colour C) pubescence density D) seed weight. **Supplementary Figure 3.** Top 20 ranked SNPs for XGBoost Seed Oil Prediction. **Supplementary Figure 4.** Top 20 ranked SNPs for XGBoost Pod Colour Prediction. **Supplementary Figure 5.** Top 20 ranked SNPs for XGBoost Seed Protein Prediction. **Supplementary Table 1.** Targeted Regions of SNPs for Reduced Input Models. **Supplementary Table 2.** List of soybean germplasm in the pangenome with the sequence coverage. (ND, not defined). **Supplementary Table 3.** Trait Data Types.

## Data Availability

The sequence metadata for all 1,110 soybean accessions are summarized in Table S2. Of these lines, 118 were previously published in PRJNA257011 [48] and 104 were previously published in PRJNA289660 [53]. The rest of the sequenced data is publicly available from the SRA project PRJNA639876 [54].
