# Supplementary material for: Machine learning models outperform deep learning models, provide interpretation and facilitate feature selection for soybean trait prediction
Source: BMC Plant Biol. 2022 Apr 8;22:180. doi: 10.1186/s12870-022-03559-z (PMC8991976; doi:10.1186/s12870-022-03559-z)
Supplement: Supplementary file 1 — Additional file 1: Supplementary Figure 1. P-value of each SNPs association for a) flower colour b) seed coat colour c) pod colour in the soybean VCF. SNPs coloured red have been determined as significantly associated for the given trait as they have a p-value less than the -log10(8) significance threshold for this GWAS. Supplementary Figure 2. Graphs ranking the top 20 most input SNPs by gain as identified by XGBoost models for trait predictions for traits with regions of importance identified from XGBoost. Blue bars are region of importance, whereas other colours represent collections of important SNPs on the same chromosome. Black bars represent left over SNPs with no relation to other SNPs in the ranking. SNP rankings for genome wide SNP input for A) flower colour B) seed coat colour C) pubescence density D) seed weight. Supplementary Figure 3. Top 20 ranked SNPs for XGBoost Seed Oil Prediction. Supplementary Figure 4. Top 20 ranked SNPs for XGBoost Pod Colour Prediction. Supplementary Figure 5. Top 20 ranked SNPs for XGBoost Seed Protein Prediction. Supplementary Table 1. Targeted Regions of SNPs for Reduced Input Models. Supplementary Table 2. List of soybean germplasm in the pangenome with the sequence coverage. (ND, not defined). Supplementary Table 3. Trait Data Types. [file 12870_2022_3559_MOESM1_ESM.zip › Suppl_table_1_targeted_regions.pdf]

**Supplementary Table 1. Targeted Regions of SNPs for Reduced Input Models**

| Trait | Flower Colour | Pod Colour | Pubescence Density |
|---|---|---|---|
| Targeted Regions | GM13 16M-19M | GM01 54.5M-55.5M | GM01 58M-59M |
| | | GM02 41.5M-42.5M | GM08 0.0M-0.5M |
| | | GM03 0.5M-1M | GM12 37M-38M |
| | | GM05 11.5M-12.5M | |
| | | GM05 6M-7M | |
| | | GM07 17M-18M | |
| | | GM07 42M-46M | |
| | | GM09 4.5M-7M | |
| | | GM19 39M-40M | |

| Seed Coat Colour | Seed Oil % | Seed Protein % | Seed Weight |
|---|---|---|---|
| GM02 44M-45M | GM01 54M-55M | GM08 8.5M-9.5M | GM03 38M-39.5M |
| GM06 48M-49M | GM08 45M-50M | GM11 11M-12M | GM11 2M-4.5M |
| GM08 7.5M-9.5M | GM11 14M-17M | GM16 32M-33M | GM13 20M-21M |
| GM12 12M-16M | GM19 52M-53M | GM20 32.5M-34M | GM18 3M-4M |
| GM19 39M-40M | GM20 8M-9M | | |
