# Supplementary material for: Machine learning models outperform deep learning models, provide interpretation and facilitate feature selection for soybean trait prediction
Source: BMC Plant Biol. 2022 Apr 8;22:180. doi: 10.1186/s12870-022-03559-z (PMC8991976; doi:10.1186/s12870-022-03559-z)
Supplement: Supplementary file 1 — Additional file 1: Supplementary Figure 1. P-value of each SNPs association for a) flower colour b) seed coat colour c) pod colour in the soybean VCF. SNPs coloured red have been determined as significantly associated for the given trait as they have a p-value less than the -log10(8) significance threshold for this GWAS. Supplementary Figure 2. Graphs ranking the top 20 most input SNPs by gain as identified by XGBoost models for trait predictions for traits with regions of importance identified from XGBoost. Blue bars are region of importance, whereas other colours represent collections of important SNPs on the same chromosome. Black bars represent left over SNPs with no relation to other SNPs in the ranking. SNP rankings for genome wide SNP input for A) flower colour B) seed coat colour C) pubescence density D) seed weight. Supplementary Figure 3. Top 20 ranked SNPs for XGBoost Seed Oil Prediction. Supplementary Figure 4. Top 20 ranked SNPs for XGBoost Pod Colour Prediction. Supplementary Figure 5. Top 20 ranked SNPs for XGBoost Seed Protein Prediction. Supplementary Table 1. Targeted Regions of SNPs for Reduced Input Models. Supplementary Table 2. List of soybean germplasm in the pangenome with the sequence coverage. (ND, not defined). Supplementary Table 3. Trait Data Types. [file 12870_2022_3559_MOESM1_ESM.zip › suppl_table_2_accesssions_meta_data.pdf]

Supplementary Table 2. List of soybean germplasm in the pangenome with the sequence coverage. (ND, not defined)

Data-storage-ID	PI-ID	Estimated coverage (Gbp)	Species	Group	Origin country	Origin	Collection year	Plant name	Gene number	Maturity group
AB-01	PI458020	19.90	*G. max*	Landrace	Korea	Seoul	1981	KAS 100-11-2	48643	IV
AB-02	PI603713	27.95	*G. max*	Landrace	China	Sichuan	1998	ZDD12392	48762	V
BR-01	PI628809	21.22	*G. max*	Modern cultivar	Brazil	ND	2001	BR-16	48913	VI
BR-02	BRS 232	12.83	*G. max*	Modern cultivar	Brazil	ND	ND	ND	48800	VII
BR-03	PI675651	17.81	*G. max*	Modern cultivar	Brazil	ND	2015	BRS 284	48878	VI
BR-04	BRS 360RR	20.91	*G. max*	Modern cultivar	Brazil	ND	ND	ND	48867	VI
BR-05	PI675666	19.87	*G. max*	Modern cultivar	Brazil	ND	2015	BRS Sambaiba	48818	VII
BR-06	PI675660	20.07	*G. max*	Modern cultivar	Brazil	ND	2016	BRS 361	48905	IX
BR-07	BRS Valiosa RR	16.24	*G. max*	Modern cultivar	Brazil	ND	ND	ND	48784	VIII
BR-08	BRSGO 8660	14.50	*G. max*	Modern cultivar	Brazil	ND	ND	ND	48840	VIII
BR-09	BRSMG 850 GRR	22.64	*G. max*	Modern cultivar	Brazil	ND	ND	ND	48812	VIII
BR-10	BRSMT Pintado	19.92	*G. max*	Modern cultivar	Brazil	ND	ND	ND	48875	VIII
BR-11	CD 201	23.09	*G. max*	Modern cultivar	Brazil	ND	ND	ND	48881	VI
BR-12	PI663948	21.46	*G. max*	Modern cultivar	Brazil	ND	2011	Embrapa 48	48893	VII
BR-13	PI628838	19.20	*G. max*	Modern cultivar	Brazil	ND	2001	FT-Abyara	48861	VII
BR-14	PI628808	23.22	*G. max*	Modern cultivar	Brazil	ND	2001	BR-14 (Modelo)	48879	VIII
BR-15	IAC 8	21.17	*G. max*	Modern cultivar	Brazil	ND	ND	ND	48855	VIII
BR-16	IAS 5	15.67	*G. max*	Modern cultivar	Brazil	ND	ND	ND	48790	VI
BR-17	MG/BR 46	28.57	*G. max*	Modern cultivar	Brazil	ND	ND	ND	48870	VIII
BR-18	NA 5909 RG	19.82	*G. max*	Modern cultivar	Brazil	ND	ND	ND	48824	VI
BR-20	PI628879	17.16	*G. max*	Modern cultivar	Brazil	ND	2001	Parana	48856	V
BR-23	PI628910	11.75	*G. max*	Modern cultivar	Brazil	ND	2001	BR-23	48673	V

BR-24	PI628911	10.17	*G. max*	Modern cultivar	Brazil	ND	2001	BR-24	48740	V
BR-29	BRSMT Uirapuru	10.29	*G. max*	Modern cultivar	Brazil	ND	ND	ND	48707	IX
BR-30	PI628925	12.93	*G. max*	Modern cultivar	Brazil	ND	2001	Embrapa-20 (DokoRC)	48781	VIII
BR-32	BRSGO Chapadões	12.86	*G. max*	Modern cultivar	Brazil	ND	ND	ND	48699	IX
DT2000	PI635999	17.34	*G. max*	Modern cultivar	Vietnam	ND	2004	DT 2000	48249	VI
ESS	PI548667	46.76	*G. max*	Modern cultivar	USA	Virginia	1972	Essex	48707	V
For	PI548645	25.51	*G. max*	Modern cultivar	USA	Illinois	1989	Pharaoh	48455	IV
HN001	PI518664	16.30	*G. max*	Modern cultivar	USA	Virginia	1988	Hutcheson	48187	V
HN002	PI548402	17.36	*G. max*	Old cultivar	China	Beijing	1954	Peking	48324	IV
HN003	PI89772	18.16	*G. max*	Landrace	China	ND	1930	7193	48363	IV
HN004	PI90763	16.07	*G. max*	Landrace	China	Beijing	1930	7570	48252	IV
HN005	PI404166	18.37	*G. max*	Landrace	China	ND	1975	VIR 5222	48349	III
HN006	PI407788A	17.02	*G. max*	Landrace	Korea	Kyonggi	1975	ORD 8113	48324	IV
HN007	PI424298	17.92	*G. max*	Landrace	Korea	Chungcheongbuk-do	1978	KAS 300-10	48355	IV
HN008	PI437655	15.16	*G. max*	Landrace	China	ND	1979	VIR 5269	48333	III
HN009	PI495017C	17.35	*G. max*	Landrace	China	Beijing	1985	Beijing da qing don	48309	IV
HN010	PI468915	13.76	*G. max*	Landrace	China	Liaoning	1982	ND	48327	II
HN011	PI507354	14.79	*G. max*	Landrace	Japan	Hokkaido	1987	Tokei 421	48244	I
HN012	PI567305	14.77	*G. max*	Landrace	China	Gansu	1993	Hei dou zi	48382	IV
HN013	S05-11482	17.39	*G. max*	Modern cultivar	USA	ND	ND	ND	48168	V
HN015	PI437654	17.87	*G. max*	Landrace	China	ND	1980	VIR 5268	48420	III
HN016B	PI567387	13.49	*G. max*	Landrace	China	Shanxi	1993	Huang huai dou	48312	IV
HN017B	PI437725	13.56	*G. max*	Landrace	China	ND	1980	VIR 5430	48278	IV
HN018	PI437690	15.96	*G. max*	Landrace	China	ND	1980	VIR 5283	48308	III
HN019	PI548402-2	18.08	*G. max*	Old cultivar	China	Beijing	1954	Peking	48382	IV
HN021	PI209332	17.39	*G. max*	Landrace	Japan	Hokkaido	1953	No.4	48392	IV
HN022	PI404198B	16.48	*G. max*	Landrace	China	ND	1975	VIR 5518	48411	IV
HN023	PI424608A	13.89	*G. max*	Landrace	Korea	Kyongsang Puk	1978	KAS 681-21	48193	IV

HN024	PI548316	19.43	*G. max*	Old cultivar	China	Zhejiang	1947	Cloud	48401	III
HN025	PI567516C	14.78	*G. max*	Landrace	China	Shandong	1993	Ba yue zha	48400	IV
HN026	PI612611	16.58	*G. max*	Landrace	Korea	ND	1998	Browngilgun	48362	III
HN027	S10-11227	18.55	*G. max*	Modern cultivar	USA	ND	ND	ND	48233	V
HN028	PI572239	13.46	*G. max*	Modern cultivar	USA	North Carolina	1993	Holladay	48076	V
HN029	IA3023	13.53	*G. max*	Modern cultivar	USA	ND	ND	ND	48131	IV
HN030	PI598124	13.53	*G. max*	Modern cultivar	USA	Missouri	1997	Maverick	48173	III
HN031	PI79691-4	13.54	*G. max*	Landrace	China	Heilongjiang	1929	ND	48281	III
HN032	PI86006	13.53	*G. max*	Landrace	Japan	Hokkaido	1930	Kiio Shokuzu	48359	III
HN033	PI87617	13.54	*G. max*	Landrace	Korea	Hamkysongpukto	1930	Miyongaikon	48202	III
HN034	PI87631-1	13.46	*G. max*	Landrace	Japan	Saitama	1930	Kindaizu	48251	III
HN035	PI196175	13.54	*G. max*	Landrace	Korea	South	1947	ND	48230	V
HN036	PI200471	14.29	*G. max*	Landrace	Japan	Shikoku	1952	Hanayome Ibaragi No. 1	48279	III
HN037	PI200508	13.37	*G. max*	Landrace	Japan	Shikoku	1952	Natsu Daizu	48214	I
HN038	PI248515	13.55	*G. max*	Landrace	Japan	Honshu	1958	White Hilum Iwata Variety No. 2	48409	IV
HN039	PI366121	13.53	*G. soja*	Wild soybean	Japan	Aizutakada	1971	ND	48611	IV
HN040	PI378702	13.48	*G. soja*	Wild soybean	Japan	Iwate	1973	ND	48502	IV
HN041	PI398593	13.28	*G. max*	Landrace	Korea	Chungcheongbuk-do	1974	KAS 390-4	48300	V
HN042	PI398595	13.34	*G. max*	Landrace	Korea	Chungcheongbuk-do	1974	KAS 390-5	48226	V
HN043	PI398610	13.32	*G. max*	Landrace	Korea	Chungcheongbuk-do	1974	KAS 390-8	48246	V
HN044	PI398614	13.28	*G. max*	Landrace	Korea	Chungcheongbuk-do	1974	KAS 390-9	48223	V
HN045	PI407162	13.50	*G. soja*	Wild soybean	Korea	Kyonggi	1972	K1-D	48518	IV
HN046	PI407184	13.53	*G. soja*	Wild soybean	Korea	Hwaseong Gun	1972	K29	48529	IV
HN047	PI407729	13.52	*G. max*	Landrace	China	Beijing	1974	ND	48275	IV
HN048	PI407965	13.56	*G. max*	Landrace	Korea	Cholla Nam	1975	KAERI 504-4	48310	V
HN049	PI408105A	13.30	*G. max*	Landrace	Korea	Kyongsang Puk	1975	KAS 633-19	48249	IV

HN050	PI416937	15.28	*G. max*	Landrace	Japan	Kanto	1974	Houjaku Kuwazu	48219	VI
HN051	PI424078	13.56	*G. max*	Landrace	Korea	Kangwon	1976	74077	48377	III
HN052	PI424079	13.52	*G. soja*	Wild soybean	Korea	Jung-sun Gun	1974	74079	48548	IV
HN053	PI424088	13.76	*G. soja*	Wild soybean	Korea	Je-chun Gun	1976	74088	48560	IV
HN054	PI437169B	15.10	*G. max*	Landrace	Russia	Krasnodar	1979	VNIISC-4	48246	II
HN055	PI437679	13.30	*G. max*	Landrace	China	ND	1979	VIR 4895	48301	IV
HN056	PI437863A	13.52	*G. max*	Landrace	China	Northeast	1979	DV-2841	48219	II
HN057	PI438258	13.57	*G. max*	Landrace	China	Northeast	1979	VIR 4714	48305	II
HN058	PI458515	13.67	*G. max*	Landrace	China	Shandong	1981	Tie Zhugan	48443	IV
HN059	PI464920B	13.71	*G. max*	Landrace	China	Liaoning	1982	Jin Dou 33	48228	III
HN060	PI467312	13.53	*G. max*	Landrace	China	Jilin	1982	Cha-mo-shi-dou	48297	II
HN061	PI471938	13.51	*G. max*	Landrace	Nepal	ND	1982	197	48219	V
HN062	PI475783B	13.45	*G. max*	Landrace	China	ND	1982	Tsing 2	48154	III
HN063	PI483463	13.40	*G. soja*	Wild soybean	China	Shanxi	1984	ND	48510	III
HN064	PI518751	13.64	*G. max*	Modern cultivar	Former Serbia	ND	1988	NS-20	48167	II
HN065	PI542044	15.48	*G. max*	Modern cultivar	USA	Illinois	1990	Kunitz	48164	III
HN066	PI547862	13.49	*G. max*	Modern cultivar	USA	Illinois	1989	L83-570	48170	III
HN067	PI548317	15.98	*G. max*	Old cultivar	China	Hebei	1954	Columbia	48260	III
HN068	PI548349	13.50	*G. max*	Old cultivar	Korea	Phyeongyang	1927	Ilsoy	48221	III
HN069	PI548415	13.42	*G. max*	Old cultivar	China	Zhejiang	1907	Sooty	48248	IV
HN070	PI548511	13.44	*G. max*	Modern cultivar	USA	Indiana	1978	Beeson 80	48146	II
HN071	PI548657	13.70	*G. max*	Modern cultivar	USA	North Carolina	1953	Jackson	48208	VII
HN072	PI549031	13.71	*G. max*	Landrace	China	Beijing	1990	ND	48282	III
HN073	PI552538	13.73	*G. max*	Modern cultivar	USA	Nebraska	1991	Dunbar	48160	III
HN074	PI561271	13.46	*G. max*	Landrace	China	Zhejiang	1992	Pei xian da quing dou	48199	V
HN075	PI567230	13.83	*G. max*	Landrace	China	Shanxi	1993	WJK-PRC-23	48316	V
HN076	PI567336B	13.79	*G. max*	Landrace	China	Gansu	1993	Lao hei dou	48335	IV
HN077	PI567343	13.80	*G. max*	Landrace	China	Gansu	1993	Ma huang dou	48337	V

HN078	PI567354	13.83	*G. max*	Landrace	China	Gansu	1993	You huang dou	48267	IV
HN079	PI567357	13.78	*G. max*	Landrace	China	Ningxia	1993	Du jia qiao huang dou	48249	III
HN080	PI567383	13.81	*G. max*	Landrace	China	Shanxi	1993	Da ke huang dou	48299	V
HN081	PI567519	13.58	*G. max*	Landrace	China	Shandong	1993	Bai hua chi	48268	III
HN082	PI567611	13.56	*G. max*	Landrace	China	Henan	1993	Ba yue zha	48241	IV
HN083	PI567651	13.53	*G. max*	Landrace	China	Henan	1993	Shang cai er cao PIng ding shi	48196	IV
HN084	PI567690	13.58	*G. max*	Landrace	China	Anhui	1993	Fuyang 7	48272	III
HN085	PI567719	13.59	*G. max*	Landrace	China	Anhui	1993	Fuyang 43	48255	IV
HN086	PI567731	13.57	*G. max*	Landrace	China	Anhui	1993	Fu yang 56	48238	III
HN087	PI591539	13.48	*G. max*	Modern cultivar	USA	Illinois	1995	L91-8558	48162	III
HN088	PI593258	13.59	*G. max*	Modern cultivar	USA	Illinois	1996	Macon	48171	III
HN089	PI594012	13.55	*G. max*	Landrace	Korea	South	1995	Heuksa tangdu	48249	V
HN090	PI594512A	16.00	*G. max*	Landrace	China	Sichuan	1996	Bian zi jiang se dou	48234	VII
HN091	PI594599	13.55	*G. max*	Landrace	China	Hunan	1996	Chang de chun hei dou	48368	IV
HN092	PI597387	13.52	*G. max*	Modern cultivar	USA	Illinois	1997	Pana	48544	III
HN093	PI603154	13.64	*G. max*	Landrace	Korea	ND	1997	GL 2622 /96	48244	V
HN094	PI603170	13.57	*G. max*	Landrace	Korea	ND	1997	GL 2683 /96	48152	IV
HN095	PI603175	16.18	*G. max*	Landrace	Korea	ND	1997	GL 2688 /96	48256	IV
HN096	PI603176A	13.62	*G. max*	Landrace	Korea	ND	1997	ND	48197	IV
HN097	PI603497	13.56	*G. max*	Landrace	China	Shandong	1998	Hua dou	48186	III
HN098	PI605869A	13.41	*G. max*	Landrace	Vietnam	Laocai	1998	Sample 140	48206	V
HN099	PI639740	13.73	*G. max*	Modern cultivar	USA	Illinois	2005	LD00-3309	48104	IV
HN100	PI647086	13.63	*G. max*	Modern cultivar	USA	North Carolina	2007	N8001	48126	VIII
HN101	PI658519	13.61	*G. max*	Modern cultivar	USA	Illinois	2009	LD00-2817P	48174	IV
HN104	FC31721	14.92	*G. max*	Landrace	ND	ND	ND	ND	48264	VI

HN105	PI438471	14.94	*G. max*	Landrace	Sweden	Ostergotlands	1947	Fiskeby III	48254	00
HN106	PI417091	15.41	*G. max*	Landrace	Japan	Kanto	1974	Kuro mame	48350	II
HN107	PI417015	14.52	*G. max*	Landrace	Japan	Iwate	1974	Kawanagare (Iwate)	48269	III
HN108	PI229343	14.18	*G. max*	Landrace	Japan	ND	1955	ND	48273	IV
HNEX-01	FC4007B	20.05	*G. max*	Landrace	ND	ND	1924	ND	48573	III
HNEX-02	PI68508	25.77	*G. max*	Landrace	China	ND	1926	292	48592	II
HNEX-04	PI68600	23.59	*G. max*	Landrace	China	Jilin	1926	382	48556	II
HNEX-06	PI80047 1	24.52	*G. max*	Landrace	Japan	Tokyo	1929	Sousei Kuro Sakigake	48591	II
HNEX-07	PI86050	21.93	*G. max*	Landrace	Japan	Hokkaido	1930	Rasuto San	48582	II
HNEX-08	PI90566-1	24.19	*G. max*	Landrace	China	Liaoning	1930	6476	48619	III
HNEX-09	PI91730-1	22.75	*G. max*	Landrace	China	Jilin	1931	Excellent, Grade No. 1	48681	III
HNEX-10	PI189930	22.81	*G. max*	Landrace	France	ND	1950	Mandchurische	48663	II
HNEX-11	PI227328	22.11	*G. max*	Landrace	Japan	Hokkaido	1955	Manchikin	48638	III
HNEX-13	PI253665D	23.16	*G. max*	Landrace	China	ND	ND	ND	48828	III
HNEX-14	PI283331	22.09	*G. max*	Landrace	Morocco	ND	1962	No. 380	48751	III
HNEX-16	PI297515	19.83	*G. max*	Landrace	Hungary	ND	1964	Iregi GM/1	48555	II
HNEX-17	PI297544	21.75	*G. max*	Landrace	Russia	Primorye	1964	ND	48597	II
HNEX-18	PI361064	21.59	*G. max*	Landrace	Romania	ND	1971	F. 56-51	48673	II
HNEX-19	PI361066A	21.53	*G. max*	Landrace	Romania	ND	1971	F. 56-17	48581	I
HNEX-20	PI370059	22.82	*G. max*	Landrace	Russia	ND	1972	VIR 5622	48638	I
HNEX-21	PI384469A	26.29	*G. max*	Landrace	Russia	Krasnodar	1974	Kubanskaja 33	48663	I
HNEX-23	PI391594	23.32	*G. max*	Landrace	China	Jilin	1974	Jilin No. 8	48531	II
HNEX-24	PI404157	27.11	*G. max*	Landrace	Russia	Primorye	1975	Primorskaja 494	48581	I
HNEX-25	PI407710	19.77	*G. max*	Landrace	China	Heilongjiang	1975	Foo sun No. 1	48564	I
HNEX-26	PI407720	20.59	*G. max*	Landrace	China	Jilin	1975	Kao chien tao	48503	II
HNEX-27	PI424159B	22.41	*G. max*	Landrace	Korea	Kyongsang Puk	1978	KAS 643-8	48447	IV
HNEX-28	PI424195B	24.33	*G. max*	Landrace	Hungary	ND	1978	ISZ-3	48499	I
HNEX-29	PI427099	21.94	*G. max*	Landrace	China	Jilin	1978	Jilin No. 3	48524	I
HNEX-30	PI436682	25.06	*G. max*	Landrace	China	Jilin	1979	Ji Lin 15	48660	I
HNEX-32	PI437851A	26.69	*G. max*	Landrace	China	Northeast	1979	DV-2369/2	48658	I
HNEX-33	PI438151	22.55	*G. max*	Landrace	China	Northeast	1980	VIR 2921	48543	I

HNEX-34	PI438206	20.14	*G. max*	Landrace	China	Northeast	1979	VIR 4492	48632	I
HNEX-35	PI445830	20.22	*G. max*	Landrace	Romania	ND	1980	Flora	48530	I
HNEX-36	PI445837	18.98	*G. max*	Landrace	Romania	ND	1980	Violeta	48581	I
HNEX-37	PI458511	20.16	*G. max*	Landrace	China	Liaoning	1981	Kai Yu No. 3	48557	II
HNEX-38	PI468377	17.62	*G. max*	Landrace	China	Heilongjiang	1982	Hong Kong #3	48514	00
HNEX-39	PI561319A	27.00	*G. max*	Landrace	China	Beijing	1992	Hui nan zi hua he jia	48587	II
HNEX-40	PI561377	25.74	*G. max*	Landrace	Japan	Natto	1992	Chohakuzan	48642	II
HNSM-01	PI567139B	23.28	*G. max*	Landrace	Indonesia	Bali	1993	MARIF 2810	48634	ND
HNSM-02	PI407985	26.59	*G. max*	Landrace	Korea	Cholla Nam	1975	KAS 542-4	48635	IV
HNSM-03	PI408319C	21.03	*G. max*	Landrace	Korea	kyongsan	1975	KAS 634-4	48645	IV
HNSM-04	PI157432	23.83	*G. max*	Landrace	Korea	South	1947	Ic san	48612	V
HNSM-05	PI200543	22.23	*G. max*	Landrace	Japan	Shikoku	1952	Tamanishiki	48538	VII
HNSM-06	PI200553	24.66	*G. max*	Landrace	Japan	Shikoku	1952	Zairai Kuro Daizu	48702	VI
HNSM-07	PI398666	22.79	*G. max*	Landrace	Korea	Chungcheongbuk-do	1972	KAS 391-4	48553	IV
HNSM-08	PI398775	22.54	*G. max*	Landrace	Korea	Chungcheongbuk-do	1972	KAS 352-4	48543	IV
HNSM-09	PI398791	24.81	*G. max*	Landrace	Korea	Chungcheongnamdo	1972	KAS 353-7	48656	IV
HNSM-10	PI398946	27.44	*G. max*	Landrace	Korea	Cholla Nam	1973	KLS 607-3	48525	IV
HNSM-11	PI399004	24.68	*G. max*	Landrace	Korea	Kyongsang Puk	1967	KLS 733-1	48553	IV
HNSM-12	PI424237A	22.98	*G. max*	Landrace	Korea	Kyonggi	1978	ND	48596	IV
HNSM-13	PI424237B	24.01	*G. max*	Landrace	Korea	Kyonggi	1978	ND	48595	VI
HNSM-14	PI424477	24.66	*G. max*	Landrace	Korea	kyongsan	1978	KAS 603-5	48533	IV
HNSM-15	PI398440	22.16	*G. max*	Landrace	Korea	Kangwon	1972	KAS 205-11	48545	IV
HNSM-16	PI399079	24.02	*G. max*	Landrace	Korea	Chungcheongbuk-do	1967	ND	48596	IV
HNSM-17	PI408029	26.30	*G. max*	Landrace	Korea	Cholla Nam	1975	KAS 570-1	48617	IV
HNSM-18	PI408097	22.02	*G. max*	Landrace	Korea	Kyongsang Puk	1975	KAS 633-11	48582	IV
HNSM-19	PI408111	24.03	*G. max*	Landrace	Korea	Kyongsang Puk	1975	ND	48682	IV
HNSM-20	PI408132	20.91	*G. max*	Landrace	Korea	Kyongsang Puk	1975	KAS 640-1	48541	IV
HNSM-21	PI273483D	21.51	*G. max*	Landrace	Korea	Seoul	1961	ND	48627	IV
HNSM-22	V71-370	23.89	*G. max*	Landrace	ND	ND	ND	ND	48672	IV

HNSM-23	PI427106	23.52	*G. max*	Landrace	China	Jilin	1976	Kung chiao 6602-3	48710	II
HNSM-24	PI427105B	24.12	*G. max*	Landrace	China	Jilin	ND	Little Golden Yellow No. 1	48575	II
HNSM-25	PI525453	21.34	*G. max*	Modern cultivar	USA	Iowa	1988	Conrad	48538	II
HNSM-26	PI548616	24.94	*G. max*	Modern cultivar	USA	Iowa	1978	Sloan	48644	II
HNSM-27	PI407076	22.98	*G. soja*	Wild soybean	Japan	Aiti	1972	RB 1072	48807	ND
HNSM-28	PI507582	24.52	*G. soja*	Wild soybean	Japan	Aomori	1983	NIAR 020001	48902	ND
HNSM-29	PI507597	24.29	*G. soja*	Wild soybean	Japan	Niigata	1984	NIAR 030005	48883	ND
HNSM-30	PI507621	23.69	*G. soja*	Wild soybean	Japan	Nagano	1983	NIAR 040019	48930	ND
HNSM-31	PI507794	22.40	*G. soja*	Wild soybean	Russia	Primorye	1986	VIR 9037	48967	ND
HNSM-32	PI562539	24.60	*G. soja*	Wild soybean	Korea	Kyonggi	1986	KA16	48904	ND
HNSM-33	PI562547	22.64	*G. soja*	Wild soybean	Korea	Chungcheongbuk-do	1986	KC1	48899	ND
HNSM-34A	Unknown	24.11	*Unknown*	Unknown	ND	ND	ND	ND	48604	ND
HNSM-35a	Unknown	23.39	*Unknown*	Unknown	ND	ND	ND	ND	48608	ND
HNY-01	R11-7999	24.15	*G. max*	Modern cultivar	USA	ND	ND	ND	48099	V
HNY-02	R10-5828	23.83	*G. max*	Modern cultivar	USA	ND	ND	ND	48072	V
HNY-03	R02-6268	25.04	*G. max*	Modern cultivar	USA	ND	ND	ND	48198	V
HNY-04	R08-1450	24.01	*G. max*	Modern cultivar	USA	ND	ND	ND	48163	V
HNY-05	R05-655	24.26	*G. max*	Modern cultivar	USA	ND	ND	ND	48244	V
HNY-06	R07-2000	23.01	*G. max*	Modern cultivar	USA	ND	ND	ND	48144	V
HNY-07	PI567425B	23.88	*G. max*	Landrace	China	Shanxi	1993	Bai hua huang dou	48291	III
HNY-08	PI597473	24.55	*G. max*	Landrace	China	Hubei	1997	82-24	48215	V
HNY-09	PI567671A	24.38	*G. max*	Landrace	China	Henan	1993	Yan ling xiao li huang	48244	III
HNY-10	PI567614B	22.14	*G. max*	Landrace	China	Henan	1993	Chang yuan xiao tian e dan	48222	IV

HNY-11	SS98-3403	21.85	*G. max*	Modern cultivar	USA	ND	ND	ND	48119	IV
HNY-12	LG11-8737	20.08	*G. max*	Modern cultivar	USA	ND	ND	ND	48235	IV
HNY-13	LG11-8744	25.06	*G. max*	Modern cultivar	USA	ND	ND	ND	48214	IV
HNY-14	PI483464 A	24.64	*G. soja*	Wild soybean	China	Ningxia	1980	ND	48477	III
HNY-15	S12-1879	23.45	*G. max*	Modern cultivar	USA	ND	ND	ND	48121	IV
HNY-16	S12-2894	22.19	*G. max*	Modern cultivar	USA	ND	ND	ND	48144	IV
HNY-17	S09-9943	22.08	*G. max*	Modern cultivar	USA	ND	ND	ND	48170	IV
HNY-18	S12-2994M	26.34	*G. max*	Modern cultivar	USA	ND	ND	ND	48134	IV
HNY-19	S12-3187	20.11	*G. max*	Modern cultivar	USA	ND	ND	ND	48114	IV
HNY-20	S11-17025	23.46	*G. max*	Modern cultivar	USA	ND	ND	ND	48098	IV
HNY-21	S11-16195	24.86	*G. max*	Modern cultivar	USA	ND	ND	ND	48115	IV
HNY-22	S08-17361	23.49	*G. max*	Modern cultivar	USA	ND	ND	ND	48188	IV
HNY-23	S09-13185	26.27	*G. max*	Modern cultivar	USA	ND	ND	ND	48127	IV
HNY-24	OSAGE	19.11	*G. max*	Modern cultivar	USA	ND	ND	ND	48100	ND
HNY-25	ANAND	21.72	*G. max*	Modern cultivar	USA	Missouri	2000	Anand	48073	ND
HNY-26	STODDARD	27.08	*G. max*	Modern cultivar	USA	ND	ND	ND	48150	ND
HNY-27	JTN 5503	20.69	*G. max*	Modern cultivar	USA	ND	ND	ND	48111	ND
HNY-28	560 IT	23.27	*G. max*	Modern cultivar	USA	ND	ND	ND	48188	ND
HNY-29	S07-5049	22.65	*G. max*	Modern cultivar	USA	ND	ND	ND	48103	IV
HNY-30	S07-5117	23.60	*G. max*	Modern cultivar	USA	ND	ND	ND	48211	IV
HNY-31	S07-6699	23.52	*G. max*	Modern cultivar	USA	ND	ND	ND	48201	IV
HNY-32	PI654356	21.16	*G. max*	Modern cultivar	USA	Missouri	2008	S99-2281	48113	IV
HNY-33	PI595645	24.39	*G. max*	Modern cultivar	USA	Georgia	1996	Benning	48183	VII
HNY-38	LG04-5196	26.60	*G. max*	Modern cultivar	USA	ND	ND	ND	48188	IV

HNY-39	LG04-6000	19.95	*G. max*	Modern cultivar	USA	ND	ND	ND	48140	IV
HNY-40	U98-311442	23.03	*G. max*	Modern cultivar	USA	ND	ND	ND	48160	III
HNY-41	PI606748	22.41	*G. max*	Modern cultivar	USA	Illinois	1998	Rend	48256	IV
HNY-42	PI340042	21.07	*G. max*	Landrace	Korea	kyongsan	1969	ND	48228	IV
HNY-43	LD04-13265	25.12	*G. max*	Modern cultivar	USA	ND	ND	ND	48219	IV
HNY-44	Woodruff	22.16	*G. max*	Modern cultivar	USA	ND	ND	ND	48134	VIII
HNY-45	PI553045	20.50	*G. max*	Modern cultivar	USA	Georgia	1991	Cook	48198	VIII
HNY-46	PI615694	23.89	*G. max*	Modern cultivar	USA	North Carolina	2001	N7001	48201	VII
HNY-47	PI602597	21.93	*G. max*	Modern cultivar	USA	Georgia	1988	Boggs	48116	VI
HNY-48	PI483460B	25.55	*G. soja*	Wild soybean	China	Liaoning	1980	ND	48577	III
HNY-49	Uram	20.91	*G. max*	Modern cultivar	Korea	ND	ND	ND	48144	ND
HNY-50	PI468916	23.98	*G. soja*	Wild soybean	China	Liaoning	1982	ND	48615	III
HNY-51	PI548352	22.61	*G. max*	Old cultivar	Korea	Hamkysongpukto	1951	Jogun	48190	III
HNY-52	PI548379	25.02	*G. max*	Old cultivar	China	Heilongjiang	1956	Mandarin (Ottawa)	48186	0
HNY-53	PI360955A	22.55	*G. max*	Landrace	Sweden	Ostergotlands	1970	ND	48342	000
HNY-54	PI653935	24.77	*G. max*	Landrace	Vietnam	ND	2008	Viet. Donor No. 90	48243	III
HNY-55	PI248399	23.05	*G. max*	Landrace	Serbia	ND	1958	Italian Early	48228	0
HNY-56	LG11-8690	21.84	*G. max*	Modern cultivar	USA	ND	ND	ND	48183	IV
HNY-57	LG11-8706	23.68	*G. max*	Modern cultivar	USA	ND	ND	ND	48317	IV
HNY-58	PI619083	21.77	*G. max*	Modern cultivar	Serbia	ND	2001	Danbaekkong	48082	V
HNY-59	PI543795	21.92	*G. max*	Modern cultivar	USA	Missouri	1990	Hartwig	48131	V
PI342622A	PI342622A	12.32	*G. soja*	Wild soybean	Russia	Primorye	1967	ND	48676	I
PI378683	PI378683	11.21	*G. soja*	Wild soybean	Japan	Nonoichi	ND	ND	48862	ND
PI378684A	PI378684A	31.54	*G. soja*	Wild soybean	Japan	Saitama	1973	ND	48871	ND
PI378684B	PI378684B	13.58	*G. soja*	Wild soybean	Japan	Saitama	1973	ND	48813	ND

PI378690	PI378690	17.43	*G. soja*	Wild soybean	Japan	Okawa	1973	ND	48620	ND
PI378696A	PI378696A	31.58	*G. soja*	Wild soybean	Japan	Simane	1970	ND	48823	ND
PI378696B	PI378696B	15.24	*G. soja*	Wild soybean	Japan	Simane	ND	ND	48749	ND
PI378698	PI378698	30.35	*G. soja*	Wild soybean	Japan	Yamanasi	1973	ND	49034	ND
PI378699A	PI378699A	16.41	*G. soja*	Wild soybean	Japan	Tokyo	ND	ND	48504	ND
PI407020	PI407020	15.28	*G. soja*	Wild soybean	Japan	Akita	1972	RB 1072	49005	ND
PI407038	PI407038	17.02	*G. soja*	Wild soybean	Japan	Akita	1972	RB 1072	48537	ND
PI407042	PI407042	12.02	*G. soja*	Wild soybean	Japan	Akita	1972	RB 1072	48608	ND
PI407052	PI407052	31.93	*G. soja*	Wild soybean	Japan	Iwate	1972	RB 1072	48959	ND
PI407077	PI407077	11.12	*G. soja*	Wild soybean	Japan	Aiti	1972	RB 1072	48675	ND
PI407083	PI407083	31.14	*G. soja*	Wild soybean	Japan	Aiti	1972	RB 1072	48801	ND
PI407085	PI407085	11.06	*G. soja*	Wild soybean	Japan	Aiti	1972	RB 1072	48684	ND
PI407096	PI407096	11.74	*G. soja*	Wild soybean	Japan	Hyogo	1972	RB 1072	48608	ND
PI407156	PI407156	11.31	*G. soja*	Wild soybean	Japan	Yamanasi	1972	RB 1072	48803	ND
PI407171	PI407171	9.63	*G. soja*	Wild soybean	Korea	Kyonggi	1972	K2-F	48454	IV
PI407175	PI407175	17.43	*G. soja*	Wild soybean	Korea	Kyonggi	1972	K4-B	48589	IV
PI407179	PI407179	10.68	*G. soja*	Wild soybean	Korea	Kyonggi	1972	K6-A	48718	ND
PI407183	PI407183	15.76	*G. soja*	Wild soybean	Korea	Kyonggi	1972	K8-B	48674	ND
PI407191	PI407191	9.64	*G. soja*	Wild soybean	Korea	Kyonggi	1972	K57	48865	ND
PI407202	PI407202	12.58	*G. soja*	Wild soybean	Korea	Kangwon	1972	K15	48852	ND
PI407214	PI407214	12.61	*G. soja*	Wild soybean	Korea	Chungcheongbuk-do	1972	K23-A	48736	ND
PI407220	PI407220	12.44	*G. soja*	Wild soybean	Korea	Chungcheongbuk-do	1972	K25-B	48650	ND
PI407228	PI407228	14.31	*G. soja*	Wild soybean	Korea	Chungcheongbuk-do	1972	K46-D	48996	ND
PI407229	PI407229	16.09	*G. soja*	Wild soybean	Korea	Chungcheongbuk-do	1972	K47-A	48830	ND

PI407231	PI407231	9.60	*G. soja*	Wild soybean	Korea	Chungcheongnamdo	1972	K47-c	48652	ND
PI407239	PI407239	11.35	*G. soja*	Wild soybean	Korea	Chungcheongnamdo	1972	K55	48659	ND
PI407240	PI407240	11.25	*G. soja*	Wild soybean	Korea	Kyongsang Puk	1972	ND	48737	ND
PI407241	PI407241	10.38	*G. soja*	Wild soybean	Korea	Kyongsang Puk	1972	K35-B	48462	ND
PI407243	PI407243	12.24	*G. soja*	Wild soybean	Korea	Kyongsang Puk	ND	ND	48760	ND
PI407262	PI407262	19.03	*G. soja*	Wild soybean	Korea	kyongsan	ND	ND	48601	ND
PI407270	PI407270	18.34	*G. soja*	Wild soybean	Korea	kyongsan	ND	ND	48602	ND
PI407281	PI407281	28.89	*G. soja*	Wild soybean	Korea	Cheju	ND	ND	48985	ND
PI407286	PI407286	29.19	*G. soja*	Wild soybean	Japan	Kanagawa	ND	ND	48897	ND
PI407287	PI407287	11.84	*G. soja*	Wild soybean	Japan	Kanagawa	ND	ND	49042	ND
PI407300	PI407300	10.72	*G. soja*	Wild soybean	China	Jiangsu	ND	ND	48603	ND
PI407303	PI407303	30.44	*G. soja*	Wild soybean	China	Jiangsu	ND	ND	48991	ND
PI407307	PI407307	19.35	*G. soja*	Wild soybean	China	Shanghai	ND	ND	48671	ND
PI407308	PI407308	32.55	*G. soja*	Wild soybean	Korea	Kyonggi	ND	ND	49039	ND
PI407315	PI407315	16.69	*G. soja*	Wild soybean	Korea	Chungcheongbuk-do	ND	ND	48574	ND
PI407318A	PI407318A	17.00	*G. soja*	Wild soybean	Korea	Chungcheongbuk-do	ND	ND	48731	ND
PI424004B	PI424004B	15.75	*G. soja*	Wild soybean	Korea	Kyonggi	ND	ND	48901	II
PI424007	PI424007	17.32	*G. soja*	Wild soybean	Korea	Kyonggi	ND	ND	49247	ND
PI424008A	PI424008A	31.02	*G. soja*	Wild soybean	Korea	Kyonggi	ND	ND	49129	ND
PI424014	PI424014	15.07	*G. soja*	Wild soybean	Korea	Kangwon	ND	ND	48551	ND
PI424025B	PI424025B	20.45	*G. soja*	Wild soybean	Korea	Kyonggi	ND	ND	49017	ND
PI424045	PI424045	12.13	*G. soja*	Wild soybean	Korea	Kyonggi	ND	ND	48682	ND
PI424070B	PI424070B	10.51	*G. soja*	Wild soybean	Korea	Kangwon	ND	ND	48762	ND

PI424083A	PI424083A	15.61	*G. soja*	Wild soybean	Korea	Kangwon	ND	ND	48889	ND
PI424102A	PI424102A	9.81	*G. soja*	Wild soybean	Korea	kyongsan	ND	ND	48797	ND
PI424107A	PI424107A	19.49	*G. soja*	Wild soybean	Korea	kyongsan	ND	ND	48588	ND
PI447003A	PI447003A	28.01	*G. soja*	Wild soybean	China	Neimongoi	ND	ND	49132	0
PI464890B	PI464890B	11.14	*G. soja*	Wild soybean	China	Jilin	ND	ND	48635	I
PI464925B	PI464925B	10.75	*G. soja*	Wild soybean	China	Liaoning	ND	ND	48748	0
PI468918	PI468918	32.50	*G. soja*	Wild soybean	China	Liaoning	ND	ND	49041	III
PI479746B	PI479746B	13.61	*G. soja*	Wild soybean	China	Jilin	ND	ND	48980	II
PI479768	PI479768	22.89	*G. soja*	Wild soybean	China	Heilongjiang	ND	ND	49008	0
PI483466	PI483466	11.60	*G. soja*	Wild soybean	China	Shandong	ND	ND	48953	ND
PI507609	PI507609	18.13	*G. soja*	Wild soybean	Japan	Totigi	ND	ND	48548	ND
PI507615	PI507615	20.53	*G. soja*	Wild soybean	Japan	Nagano	ND	ND	48543	ND
PI507618	PI507618	15.48	*G. soja*	Wild soybean	Japan	Nagano	ND	ND	48628	ND
PI507619B	PI507619B	17.39	*G. soja*	Wild soybean	Japan	Nagano	ND	ND	48528	ND
PI507624	PI507624	12.25	*G. soja*	Wild soybean	Japan	Gihu	1983	NIAR 050002	48540	ND
PI507638	PI507638	18.81	*G. soja*	Wild soybean	Japan	Hyogo	ND	ND	48649	ND
PI507641	PI507641	14.17	*G. soja*	Wild soybean	Japan	Hyogo	ND	ND	48667	ND
PI507667	PI507667	20.34	*G. soja*	Wild soybean	Japan	Kumamoto	ND	ND	48550	ND
PI507752	PI507752	32.95	*G. soja*	Wild soybean	Russia	Amur	ND	ND	48876	ND
PI507757	PI507757	11.44	*G. soja*	Wild soybean	Russia	Amur	ND	ND	48788	ND
PI507761	PI507761	15.10	*G. soja*	Wild soybean	Russia	Amur	ND	ND	48760	ND
PI507830B	PI507830B	18.23	*G. soja*	Wild soybean	Russia	Primorye	ND	ND	48510	ND
PI507847	PI507847	18.79	*G. soja*	Wild soybean	Russia	Amur	ND	ND	48591	ND
PI522209B	PI522209B	10.59	*G. soja*	Wild soybean	Russia	Primorye	ND	ND	48647	ND

PI522235B	PI522235B	13.46	*G. soja*	Wild soybean	Russia	Primorye	1988	ND	48427	ND
PI532450	PI532450	17.16	*G. soja*	Wild soybean	China	Jilin	ND	ND	48534	ND
PI549032	PI549032	11.57	*G. soja*	Wild soybean	China	Liaoning	ND	ND	48591	ND
PI549047	PI549047	19.29	*G. soja*	Wild soybean	China	Beijing	ND	Mrs. Wong's Picnic	48472	ND
PI549048	PI549048	20.33	*G. soja*	Wild soybean	China	Beijing	ND	ND	48505	ND
PI562534	PI562534	17.42	*G. soja*	Wild soybean	Korea	kyonggi	ND	ND	48571	ND
PI562551	PI562551	12.11	*G. soja*	Wild soybean	Korea	Chungcheongbuk-do	1986	KC26	48685	ND
PI562553	PI562553	10.74	*G. soja*	Wild soybean	Korea	Chungcheongbuk-do	1986	ND	48577	ND
PI562557	PI562557	15.40	*G. soja*	Wild soybean	Korea	Cholla	1986	KE10	48523	ND
PI562558	PI562558	29.98	*G. soja*	Wild soybean	Korea	Cholla Puk	1986	KE12	48947	ND
PI562564	PI562564	13.49	*G. soja*	Wild soybean	Korea	Cholla Puk	1992	ND	48379	ND
PI562568	PI562568	15.70	*G. soja*	Wild soybean	Korea	Cholla	1986	KF24	48521	ND
PI597448D	PI597448D	14.99	*G. soja*	Wild soybean	China	Heilongjiang	ND	ND	48557	ND
PI597458B	PI597458B	31.02	*G. soja*	Wild soybean	China	Shandong	ND	ND	49007	ND
PI597458C	PI597458C	10.00	*G. soja*	Wild soybean	China	Shandong	ND	ND	48549	ND
PI597460A	PI597460A	13.84	*G. soja*	Wild soybean	China	Shandong	1996	ND	48526	ND
PI597461B	PI597461B	13.14	*G. soja*	Wild soybean	China	Shandong	ND	ND	48790	ND
PI597462B	PI597462B	10.23	*G. soja*	Wild soybean	China	Shandong	ND	ND	48536	ND
PI597471D	PI597471D	27.84	*G. soja*	Wild soybean	China	Zhejiang	ND	ND	48901	ND
PI602450	PI602450	14.98	*G. max*	Modern cultivar	USA	Maryland	1998	BARC-15 non-nodulated	48357	IV
PI639586	PI639586	21.34	*G. soja*	Wild soybean	Russia	Amur	ND	ND	48516	ND
PI639588B	PI639588B	28.64	*G. soja*	Wild soybean	Russia	Amur	2005	KA 325	48519	ND
PI639590A	PI639590A	27.83	*G. soja*	Wild soybean	Russia	Amur	ND	ND	48756	ND

PI639590B	PI6395 90B	17.81	*G. soja*	Wild soybean	Russia	Amur	ND	ND	48448	ND
PI639621	PI6396 21	33.05	*G. soja*	Wild soybean	Russia	Amur	ND	ND	48708	ND
PI639623A	PI6396 23A	10.47	*G. soja*	Wild soybean	Russia	Amur	ND	ND	48664	ND
PI639635	PI6396 35	13.00	*G. soja*	Wild soybean	Russia	Primorye	ND	ND	48576	ND
SC-04	PI6584 98	21.88	*G. max*	Modern cultivar	USA	North Carolina	2009	N6202	48570	VI
SRR1533155	PI5625 65	18.68	*G. soja*	Wild soybean	Korea	Cholla Puk	ND	ND	49089	ND
SRR1533157	PI3935 51	13.78	*G. soja*	Wild soybean	Taiwan	Taiwan	1974	ND	48810	ND
SRR1533161	PI5939 83	18.56	*G. soja*	Wild soybean	Japan	Hokkaido	ND	ND	48910	ND
SRR1533162	PI4070 27	13.65	*G. soja*	Wild soybean	Japan	Akita	ND	ND	49044	ND
SRR1533163	PI5625 59	18.41	*G. soja*	Wild soybean	Korea	Cholla Puk	ND	ND	48928	ND
SRR1533164	PI5974 59C	16.52	*G. soja*	Wild soybean	China	Shandon g	ND	ND	48917	ND
SRR1533168	PI4071 70	29.44	*G. soja*	Wild soybean	Korea	Kyonggi	ND	ND	49412	ND
SRR1533169	PI4071 31	17.46	*G. soja*	Wild soybean	Japan	Kumamo to	ND	ND	49205	ND
SRR1533172	PI4797 52	14.12	*G. soja*	Wild soybean	China	Jilin	ND	ND	48884	I
SRR1533174	PI3661 23	17.00	*G. soja*	Wild soybean	Japan	Iwate	1971	ND	48853	IV
SRR1533175	PI4585 36	21.00	*G. soja*	Wild soybean	China	Heilongji ang	ND	ND	49206	0
SRR1533176	PI4071 97	15.25	*G. soja*	Wild soybean	Korea	Kangwon	ND	ND	48895	ND
SRR1533178	PI5221 82B	16.47	*G. soja*	Wild soybean	China	Heilongji ang	ND	ND	48903	ND
SRR1533180	PI3786 92	15.45	*G. soja*	Wild soybean	Japan	Iwate	ND	ND	48807	IV
SRR1533181	PI4649 29A	15.84	*G. soja*	Wild soybean	China	Liaoning	ND	ND	48903	0
SRR1533182	PI4072 46	16.11	*G. soja*	Wild soybean	Korea	Kyongsa ng Puk	ND	ND	49102	ND
SRR1533183	PI4649 35	17.55	*G. soja*	Wild soybean	China	Jiangsu	ND	ND	48802	ND
SRR1533185	PI5222 28	18.14	*G. soja*	Wild soybean	Russia	Primorye	ND	ND	48943	ND
SRR1533187	PI4072 75	15.84	*G. soja*	Wild soybean	Korea	Kyonggi	ND	ND	49122	IV

SRR1533188	PI464929B	16.04	*G. soja*	Wild soybean	China	Liaoning	ND	ND	49100	0
SRR1533190	PI326582A	11.24	*G. soja*	Wild soybean	Russia	Primorye	ND	ND	49270	II
SRR1533191	PI522216	15.76	*G. soja*	Wild soybean	Russia	Primorye	ND	ND	49013	ND
SRR1533192	PI468400A	17.13	*G. soja*	Wild soybean	China	Ningxia	ND	ND	48961	IV
SRR1533193	PI479769	17.99	*G. soja*	Wild soybean	China	Heilongjiang	ND	ND	48954	0
SRR1533194	PI424096	14.74	*G. soja*	Wild soybean	Korea	Chungcheongbuk-do	ND	ND	48829	ND
SRR1533196	PI504286	16.71	*G. soja*	Wild soybean	Korea	Chungcheongbuk-do	ND	ND	49158	ND
SRR1533197	PI458538	17.86	*G. soja*	Wild soybean	China	Heilongjiang	ND	ND	49001	0
SRR1533198	PI458535	15.60	*G. soja*	Wild soybean	China	Heilongjiang	ND	ND	48951	0
SRR1533199	PI407285	15.62	*G. soja*	Wild soybean	Japan	Kanagawa	1974	Yasei Daizu	48801	ND
SRR1533202	PI407288	16.54	*G. soja*	Wild soybean	China	Jilin	1974	ND	48989	II
SRR1533203	PI578341	17.65	*G. soja*	Wild soybean	Russia	Habarovsk region	1992	VIR 8512	48931	ND
SRR1533204	PI549046	21.10	*G. soja*	Wild soybean	China	Ningxia	1990	ZYD 3728	49181	ND
SRR1533205	PI464927A	17.01	*G. soja*	Wild soybean	China	Liaoning	1982	LS-005	48930	0
SRR1533206	PI483465	16.36	*G. soja*	Wild soybean	China	Shanxi	1984	ND	49072	ND
SRR1533210	PI597459D	17.90	*G. soja*	Wild soybean	China	Shandong	1996	ZYD3234	48941	ND
SRR1533211	PI597461A	21.32	*G. soja*	Wild soybean	China	Shandong	1996	ZYD3243	49383	ND
SRR1533212	PI522226	14.50	*G. soja*	Wild soybean	Russia	Far East	1988	VIR 8511	48917	ND
SRR1533216	PI594615	33.17	*G. max*	Landrace	China	Guizhou	1996	Liu yue zao	48897	IV
SRR1533220	PI196166	14.66	*G. max*	Landrace	Korea	ND	1947	No. 2296	48516	V
SRR1533221	PI467343	19.37	*G. max*	Landrace	China	Jilin	1982	Yan-nong No. 2	48597	I
SRR1533229	PI416971	14.73	*G. max*	Landrace	Japan	Kinki	1974	Kaifuu gyuumou ou 1	48738	IV
SRR1533230	PI594777	23.35	*G. max*	Landrace	China	Yunnan	1996	Liu yue huang	48439	IV
SRR1533231	PI548312	21.01	*G. max*	Old cultivar	China	Heilongjiang	1945	Cayuga	48600	I
SRR1533235	PI507355	19.71	*G. max*	Landrace	Japan	Hokkaido	1987	Tokei 423	48624	I

SRR1533236	PI587552	17.87	*G. max*	Landrace	China	Jiangsu	1995	Nan jing da PIng ding huang yi No. 1	48807	VII
SRR1533239	PI603336	40.39	*G. max*	Landrace	China	Heilongjiang	1998	Qing pi si li huang	48892	II
SRR1533243	PI602991	15.21	*G. max*	Landrace	China	Shandong	1998	Niu jiao qi da hei dou	48403	IV
SRR1533245	PI407801	10.81	*G. max*	Landrace	Korea	Kyonggi	1975	ND	48383	VI
SRR1533248	PI398296	19.67	*G. max*	Landrace	Korea	Kyonggi	1972	KAS 173-3	48636	II
SRR1533257	PI548417	11.00	*G. max*	Old cultivar	Italy	ND	1910	Soysota	48518	I
SRR1533259	PI594788	10.98	*G. max*	Landrace	China	Guizhou	1996	Liu yue zao	48701	ND
SRR1533266	PI438498	11.01	*G. max*	Landrace	USA	ND	1979	Sable	48490	IV
SRR1533269	PI153262	11.06	*G. max*	Landrace	Belgium	ND	1946	Roumanie	48374	0
SRR1533271	PI603318	13.86	*G. max*	Landrace	China	Heilongjiang	1998	Xiao zhu yao	48487	I
SRR1533272	PI437653	13.78	*G. max*	Landrace	China	ND	1979	Er-da-li	48618	II
SRR1533278	PI567189A	14.54	*G. max*	Landrace	Vietnam	ND	1993	Ekhabac	48607	IV
SRR1533279	PI548406	14.56	*G. max*	Old cultivar	China	ND	ND	ND	48601	II
SRR1533282	PI323576	11.07	*G. max*	Landrace	India	Uttar Pradesh	1967	H 67-27	49088	IX
SRR1533283	PI548382	13.41	*G. max*	Old cultivar	ND	ND	1922	Manitoba Brown	48580	0
SRR1533284	PI416890	12.86	*G. max*	Landrace	Japan	Kanto	1974	Gokuwase natsu daizu	49214	0
SRR1533285	PI587752	13.70	*G. max*	Landrace	China	Hubei	1994	Xian ning dong huang dou jia	48492	V
SRR1533286	PI424391	14.32	*G. max*	Landrace	Korea	Cholla Puk	1978	KAS 521-15	48511	VI
SRR1533288	PI603357	13.90	*G. max*	Landrace	China	Jilin	1998	Du Lu Dou	48547	I
SRR1533290	PI437944	11.93	*G. max*	Landrace	China	Northeast	1979	ND	48484	II
SRR1533292	PI157421	9.66	*G. max*	Landrace	Korea	South	1947	Ebony	48921	III
SRR1533295	PI567364	20.20	*G. max*	Landrace	China	Ningxia	1993	PIng luo huang da dou	48631	III
SRR1533297	PI548298	21.68	*G. max*	Old cultivar	China	ND	1939	A.K. (Harrow)	48491	III
SRR1533299	PI548488	19.11	*G. max*	Old cultivar	China	Heilongjiang	1938	S-100	48376	V
SRR1533301	PI423954	13.35	*G. max*	Landrace	Japan	Kumamoto	1976	Shirome	48548	0

SRR1533302	PI587848	13.92	*G. max*	Landrace	China	Hubei	1994	Wu chang hei dong dou	48477	V
SRR1533303	PI587666	22.38	*G. max*	Landrace	China	Anhui	1994	Er dao zao	48653	VI
SRR1533310	PI317334A	12.80	*G. max*	Landrace	Japan	Hokkaido	1966	Kitamishiro	48505	I
SRR1533312	PI548456	13.99	*G. max*	Old cultivar	Korea	Phyeongyang	1991	Haberland	48538	VI
SRR1533314	PI588053A	16.56	*G. max*	Landrace	China	Guangdong	ND	ND	48582	V
SRR1533315	PI594629	15.96	*G. max*	Landrace	China	Guizhou	1996	Xiao hua lian	48568	VI
SRR1533319	PI567258	12.65	*G. max*	Landrace	China	Jiangxi	1993	NC 9173	48368	II
SRR1533324	PI317336	15.30	*G. max*	Landrace	Japan	Hokkaido	1966	Shinsei	48554	0
SRR1533326	PI594579	25.16	*G. max*	Landrace	China	Hunan	1996	Zhong he tian cheng dou	48689	V
SRR1533328	PI253658B	11.44	*G. max*	Landrace	China	ND	ND	ND	48200	I
SRR1533332	PI567293	20.22	*G. max*	Landrace	China	Gansu	1993	Ben di huang dou	48806	II
SRR1533333	PI407849	16.27	*G. max*	Landrace	Korea	Cholla Puk	1975	KAS 510-1	48576	III
SRR1533336	PI89138	16.85	*G. max*	Landrace	Korea	Hamkysongpukto	1930	Zontanorukon	48486	II
SRR1533338	PI417398	14.32	*G. max*	Landrace	China	ND	1974	Touhoutorotou	48467	III
SRR1533341	PI243541	14.98	*G. max*	Landrace	Japan	Akita	1957	Shakujo	48515	IV
SRR1533345	PI548362	17.97	*G. max*	Old cultivar	USA	Illinois	1954	Lincoln	48345	III
SRR1533348	PI591495	17.45	*G. max*	Modern cultivar	USA	Illinois	1995	L93-2740	48634	IV
SRR1533351	PI548634	14.70	*G. max*	Modern cultivar	USA	Ohio	1984	Zane	48552	III
SRR1533353	PI547459	9.60	*G. max*	Modern cultivar	USA	Illinois	1975	L64-1081	48532	IV
SRR1533356	PI553047	11.28	*G. max*	Modern cultivar	USA	Georgia	1991	Gordon	48371	VII
SRR1533357	PI547562	10.72	*G. max*	Modern cultivar	USA	Illinois	1975	L72-2157	48200	IV
SRR1533362	PI533655	15.87	*G. max*	Modern cultivar	USA	Illinois	1980	Burlison	48424	II
SRR1533365	PI548638	15.47	*G. max*	Modern cultivar	Canada	Ontario	1987	OAC Libra	49019	0
SRR1533366	PI547460	16.19	*G. max*	Modern cultivar	USA	Illinois	1975	L64-1083	48546	IV
SRR1533369	PI518750	16.94	*G. max*	Modern cultivar	Former Serbia	ND	1988	NS-16	48591	I
SRR1533371	PI548540	25.01	*G. max*	Modern cultivar	USA	Iowa	1963	Corsoy	48675	II
SRR1533374	PI547686	18.83	*G. max*	Modern cultivar	USA	Illinois	1972	L62-956	48575	II

SRR1533378	PI548311	16.90	*G. max*	Old cultivar	Canada	Ontario	1988	CaPItal	48412	0
SRR1533381	PI591435	32.65	*G. max*	Modern cultivar	Canada	Ontario	1995	OT94-41	48465	ND
SRR1533384	PI548512	13.81	*G. max*	Modern cultivar	USA	Indiana	1978	Century	48506	II
SRR1533385	PI547690	15.72	*G. max*	Modern cultivar	USA	Illinois	1972	L63-1212	48333	II
SRR1533386	PI548182	20.51	*G. max*	Modern cultivar	USA	Illinois	1954	T157	48646	III
SRR1533387	PI515961	17.98	*G. max*	Modern cultivar	USA	Kentucky	1987	Pennyrile	48640	IV
SRR1533389	PI548391	21.85	*G. max*	Old cultivar	China	Liaoning	1952	Mukden	48686	II
SRR1533395	PI548631	9.91	*G. max*	Modern cultivar	USA	Illinois	1970	Williams	48366	III
SRR1533396	PI547779	9.78	*G. max*	Modern cultivar	USA	Illinois	1989	L72D-4110	48408	II
SRR1533409	PI546044	9.87	*G. max*	Modern cultivar	Canada	Ontario	1990	OT89-06	48570	ND
SRR1533415	PI548573	15.05	*G. max*	Modern cultivar	Canada	Ontario	1956	Harosoy	48491	II
SRR1533419	PI533602	14.75	*G. max*	Modern cultivar	USA	Arkansas	1989	Lloyd	48382	VI
SRR1533423	PI536635	13.20	*G. max*	Modern cultivar	USA	Ohio	1979	Sprite	48437	III
SRR1533427	PI591541	15.55	*G. max*	Modern cultivar	USA	Illinois	1995	L74-102	48443	II
SRR1533429	PI548593	15.46	*G. max*	Modern cultivar	Canada	Ontario	1980	Maple Arrow	48487	0
SRR1533436	PI591511	18.91	*G. max*	Modern cultivar	USA	Illinois	1995	L89-1581	48555	III
SRR1533438	PI513382	15.96	*G. max*	Modern cultivar	USA	Minnesota	1987	Glenwood	48673	0
SRR1533441	PI548985	16.27	*G. max*	Modern cultivar	USA	South Carolina	1991	Kershaw	48559	VI
SRR1533442	PI548477	14.79	*G. max*	Old cultivar	USA	Tennessee	1991	Ogden	49248	VI
SRR1533444	PI547680	16.12	*G. max*	Modern cultivar	USA	Illinois	1972	L62-17	48456	II
SRR1533446	PI591431	15.11	*G. max*	Modern cultivar	Canada	Ontario	1995	OT94-49	48289	ND
SRR1533451	PI542403	19.59	*G. max*	Modern cultivar	USA	Minnesota	1983	Dawson	48629	0
TC21	PI615695	56.70	*G. max*	Modern cultivar	USA	North Carolina	2001	N7103	49354	VII
TC22	PI366122	81.35	*G. soja*	Wild soybean	Japan	Hukusima	1971	ND	49794	IV
US-18	PI595099	13.36	*G. max*	Modern cultivar	USA	Georgia	1996	G93-9223	48772	VII

US-19	PI595362	16.29	*G. max*	Landrace	ND	ND	ND	ND	48196	IV
USB-001	PI548656	51.81	*G. max*	Modern cultivar	USA	Mississippi	1954	Lee	48088	VI
USB-002	FC33243	36.32	*G. max*	Landrace	ND	ND	1954	Anderson	48256	IV
USB-003	PI81785	46.82	*G. max*	Landrace	Japan	Hokkaido	1929	Chusei Hadaka	48478	III
USB-004	PI166105	45.37	*G. max*	Landrace	India	Uttar Pradesh	1948	Bhart	48514	VII
USB-005	PI180501	48.12	*G. max*	Landrace	Germany	ND	1949	Strain No. 18	48439	0
USB-006	PI240664	45.81	*G. max*	Landrace	Philippines	Luzon	1957	Bilomi No. 3	48397	ND
USB-007	PI266806C	35.87	*G. max*	Landrace	China	Hebei	1960	No. 4	48385	II
USB-009	PI360957	44.11	*G. max*	Landrace	Japan	Okhotsk Sea	1971	Karafuto No. 1	48408	0
USB-010	PI361087	44.04	*G. max*	Landrace	Romania	ND	ND	ND	48456	I
USB-011	PI361093	45.67	*G. max*	Landrace	Serbia	ND	1971	Novosadska Br. 1	48439	I
USB-012	PI437265D	45.42	*G. max*	Landrace	Moldova	ND	1979	Dobruzanca D	48516	0
USB-013	PI438335	48.25	*G. max*	Landrace	Algeria	ND	1979	SAO 196-C	48355	III
USB-014	PI468908	59.89	*G. max*	Landrace	China	Jilin	1982	ND	48633	000
USB-015	PI497964A	36.17	*G. max*	Landrace	India	Sikkim	1980	I.C. 9461	48315	IX
USB-016	PI506862	35.60	*G. max*	Landrace	Japan	Tohoku	1987	Karikei 86	48352	IV
USB-017	PI507180	45.06	*G. max*	Landrace	Japan	Kanto	1987	Rikuu 21	48390	IV
USB-018	PI518668	49.86	*G. max*	Modern cultivar	USA	Tennessee	1988	TN 4-86	48341	IV
USB-020	PI603458A	43.89	*G. max*	Landrace	China	ND	1998	Shui dou	48616	IV
USB-021	PI548171	36.63	*G. max*	Modern cultivar	USA	Illinois	1954	T134	48214	III
USB-022	PI548193	43.47	*G. max*	Modern cultivar	USA	Iowa	1957	T201	48336	IV
USB-023	PI548313	35.47	*G. max*	Old cultivar	Russia	Khabarovsk	1949	Chestnut	48318	III
USB-024	PI548325	36.37	*G. max*	Old cultivar	Russia	ND	1955	Flambeau	48365	0
USB-025	PI548360	46.35	*G. max*	Old cultivar	Korea	ND	1988	Korean	48395	II
USB-026	PI548364	42.86	*G. max*	Old cultivar	Japan	ND	1882	MacouPIn	48389	IV
USB-028	PI548447	45.70	*G. max*	Old cultivar	China	Zhejiang	1991	Cherokee	48451	VIII
USB-029	PI548452	57.61	*G. max*	Old cultivar	Korea	Phyeongyang	1991	Dixie	48500	V
USB-031	PI548490	45.00	*G. max*	Old cultivar	Taiwan	ND	1991	Tanner	48407	VII
USB-032	PI548520	44.31	*G. max*	Modern cultivar	USA	Iowa	1986	Preston	48365	II
USB-033	PI548561	45.66	*G. max*	Modern cultivar	USA	Minnesota	1973	Hodgson	48479	I
USB-035	PI549018	56.14	*G. max*	Landrace	China	Ningxia	1973	ZYD 3939	48674	V
USB-036	PI549026	51.17	*G. max*	Landrace	China	Liaoning	1990	Gao li huang	48403	V

USB-037	PI549041A	56.30	*G. max*	Landrace	China	Liaoning	1990	ZYD 2709	48569	III
USB-038	PI559932	62.04	*G. max*	Modern cultivar	USA	Maryland	1991	Manokin	48293	IV
USB-039	PI567171	52.68	*G. max*	Landrace	China	Shanxi	ND	ND	48456	0
USB-040	PI567426	55.41	*G. max*	Landrace	China	Shanxi	1993	Bai huang dou	48488	IV
USB-041	PI567558	49.35	*G. max*	Landrace	China	Shandong	1993	Liu shi ri jin huang da dou	48438	III
USB-042	PI567604A	43.02	*G. max*	Landrace	China	Shandong	1993	Xin huang dou	48449	IV
USB-043	PI578309	44.68	*G. max*	Landrace	Nepal	ND	1984	I-64	48668	VI
USB-044	PI578495	56.49	*G. max*	Landrace	China	Beijing	1994	Jin dou No. 4	48468	IV
USB-045	PI592523	45.65	*G. max*	Modern cultivar	USA	Minnesota	1995	Glacier	48408	0
USB-046	PI592954	45.65	*G. max*	Landrace	China	ND	1994	ZDD 11242	48381	II
USB-047	PI594307	56.19	*G. max*	Landrace	Japan	ND	1996	Tsurusengoku	48500	VIII
USB-048	PI597476	49.62	*G. max*	Modern cultivar	Korea	ND	1997	Deogyukong	48345	V
USB-049	PI603426G	52.99	*G. max*	Landrace	China	ND	1998	Ben di yuan huang dou	48394	II
USB-050	PI603442	51.84	*G. max*	Landrace	China	ND	1998	Ke qi xiao hei dou	48620	III
USB-052	PI88788	22.18	*G. max*	Landrace	China	Liaoning	1930	5913	48377	III
USB-058	PI548658	16.87	*G. max*	Modern cultivar	USA	Arkansas	1974	Lee 74	47982	VI
USB-059	FC29333	21.56	*G. max*	Landrace	ND	ND	1935	Laredo	48180	III
USB-060	FC31697	18.97	*G. max*	Landrace	Costa	Rica	1946	FC 31697	48105	IV
USB-061	PI54591	16.91	*G. max*	Landrace	China	Liaoning	1921	No. 31	48193	III
USB-062	PI54608-1	17.98	*G. max*	Landrace	China	Liaoning	ND	ND	48240	II
USB-063	PI54614	20.05	*G. max*	Landrace	China	Jilin	1921	No. 54	48197	IV
USB-064	PI54615-1	16.48	*G. max*	Landrace	China	Heilongjiang	ND	ND	48143	III
USB-065	PI58955	23.78	*G. max*	Landrace	China	Shandong	1924	Common Yellow Variety	48359	IV
USB-066	PI62203	24.92	*G. max*	Landrace	China	Hebei	1924	937	48318	V
USB-067	PI68521-1	16.52	*G. max*	Landrace	China	Heilongjiang	1926	205	48213	III
USB-068	PI68604-1	25.49	*G. max*	Landrace	China	ND	ND	ND	48282	III
USB-069	PI68732-1	17.00	*G. max*	Landrace	China	Heilongjiang	1926	204	48267	III
USB-070	PI70080	19.48	*G. max*	Landrace	China	Jilin	1926	6908	48267	III
USB-071	PI70466-3	26.43	*G. max*	Landrace	China	Jilin	ND	ND	48315	IV
USB-072	PI71465	18.99	*G. max*	Landrace	China	Jiangsu	1927	No. 33	48328	V

USB-073	PI80837	17.21	*G. max*	Landrace	Japan	Akita	1929	Mejiro	48215	IV
USB-074	PI81041	21.47	*G. max*	Landrace	Japan	Hokkaido	1929	Kuro Daizu	48272	III
USB-075	PI83881	21.32	*G. max*	Landrace	Korea	kangwecnto	1930	Orukon	48311	IV
USB-076	PI83942	17.56	*G. max*	Landrace	Korea	Kyonggi	1930	Kuro churyu	48295	V
USB-077	PI84631	24.22	*G. max*	Landrace	Korea	Kyonggi	1930	S-56	48300	III
USB-078	PI84637	17.90	*G. max*	Landrace	Korea	Kyonggi	ND	ND	48253	II
USB-079	PI84656	22.01	*G. max*	Landrace	Korea	Kyonggi	1930	S-81	48322	III
USB-080	PI84946-2	19.34	*G. max*	Landrace	Korea	Pusan	1929	Kandokon	48261	IV
USB-081	PI84973	19.39	*G. max*	Landrace	Japan	ND	ND	ND	48252	III
USB-084	PI86904	21.04	*G. max*	Landrace	Korea	Chungcheongbuk-do	1930	Fukota	48222	VI
USB-085	PI86972-2	26.74	*G. max*	Landrace	Korea	Cholla Puk	ND	ND	48377	IV
USB-086	PI87620	18.26	*G. max*	Landrace	Korea	Hamkysongpukto	1930	Kurome shoryu	48294	III
USB-087	PI88313	17.92	*G. max*	Landrace	China	Heilongjiang	1930	5702	48312	II
USB-088	PI88468	21.97	*G. max*	Landrace	China	Liaoning	1930	Iganzu	48342	II
USB-089	PI89005-5	16.90	*G. max*	Landrace	China	Liaoning	1930	5950	48212	II
USB-090	PI89775	18.36	*G. max*	Landrace	China	Tangshan	1930	7221	48234	VI
USB-091	PI90479P	17.03	*G. max*	Landrace	China	ND	1930	7413	48200	IV
USB-092	PI90486	18.71	*G. max*	Landrace	China	Beijing	1930	7533	48271	III
USB-093	PI91100-3	18.15	*G. max*	Landrace	China	Jilin	ND	ND	48221	III
USB-094	PI91159-4	14.08	*G. max*	Landrace	China	Liaoning	ND	ND	48207	IV
USB-095	PI91160	22.21	*G. max*	Landrace	China	Liaoning	1931	6615	48310	III
USB-096	PI92651	20.55	*G. max*	Landrace	China	Jilin	1931	7846	48198	IV
USB-097	PI94159-3	19.29	*G. max*	Landrace	Japan	kagosima	ND	ND	48288	IV
USB-098	PI95860	16.81	*G. max*	Landrace	Korea	Chungcheongbuk-do	1932	155	48199	VI
USB-100	PI103088	18.01	*G. max*	Landrace	China	Henan	1933	Ming Chuan	48283	III
USB-101	PI123440	18.62	*G. max*	Landrace	Myanmar	ND	1937	No. 2	48225	VI
USB-102	PI153231	20.28	*G. max*	Landrace	ND	ND	1946	B-63	48289	III
USB-103	PI153281	15.54	*G. max*	Landrace	Belgium	ND	1946	N-22	48245	0
USB-104	PI154189	18.17	*G. max*	Landrace	Netherlands	ND	1946	No. 57	48216	0
USB-106	PI159925	16.00	*G. max*	Landrace	Peru	Lima	ND	ND	48181	VIII
USB-107	PI165563	18.01	*G. max*	Landrace	India	Uttar Pradesh	1948	Bhart	48286	VII
USB-108	PI165675	35.18	*G. max*	Landrace	China	Jiangsu	1948	Nanking 332	48870	VII
USB-109	PI171428	16.75	*G. max*	Landrace	China	Beijing	1948	Large Yellow Soybean	48228	IV

USB-110	PI171451	13.11	*G. max*	Landrace	Japan	Kanagawa	1948	Kosamame	48178	VII
USB-112	PI179935	28.54	*G. max*	Landrace	India	Himachal Pradesh	1949	Bhart	48352	VII
USB-113	PI189873	17.99	*G. max*	Landrace	France	ND	1950	Miko Saumon	48272	0
USB-115	PI209333	17.21	*G. max*	Landrace	Japan	Hokkaido	1953	No. 3	48277	VI
USB-116	PI209334	19.14	*G. max*	Landrace	Japan	Hokkaido	ND	ND	48351	III
USB-117	PI232992	19.60	*G. max*	Landrace	Japan	Saga	1956	Kono-Kuradaizu	48351	III
USB-119	PI253661B	28.02	*G. max*	Landrace	China	ND	1958	No. 12	48359	III
USB-120	PI261272C	34.19	*G. max*	Landrace	Thailand	Nan	1959	Tua Nao	48429	IX
USB-121	PI274453	24.42	*G. max*	Landrace	Japan	Okinawa	1961	ND	48409	X
USB-122	PI291294	21.25	*G. max*	Landrace	China	Heilongjiang	1963	ND	48706	I
USB-123	PI291309D	26.30	*G. max*	Landrace	China	Heilongjiang	1963	ND	48410	II
USB-124	PI291310C	24.15	*G. max*	Landrace	China	Heilongjiang	1963	ND	48301	II
USB-125	PI297505	32.88	*G. max*	Landrace	China	ND	1964	Czi ti No. 5	48256	I
USB-126	PI297520	17.15	*G. max*	Landrace	Hungary	ND	1964	Iregi Universal	48195	0
USB-127	PI324924	21.29	*G. max*	Modern cultivar	South Africa	ND	1968	Rhosa	48178	V
USB-128	PI342434	16.73	*G. max*	Landrace	Japan	Iwate	1967	ND	48348	V
USB-129	PI342619A	15.49	*G. max*	Landrace	Russia	Primorye	1969	ND	48298	0
USB-131	PI361066B	18.81	*G. max*	Landrace	Romania	ND	1971	F. 56-17	48160	I
USB-132	PI361070	23.56	*G. max*	Landrace	Romania	ND	1971	Faur	48321	0
USB-133	PI361080	17.68	*G. max*	Landrace	Russia	ND	1971	Kormovaia 15	48325	II
USB-134	PI372403B	16.79	*G. max*	Landrace	Austria	ND	1972	Caloria	48260	0
USB-135	PI372418	17.13	*G. max*	Landrace	Serbia	ND	1972	Novosadska Br. 4	48244	I
USB-136	PI374207	16.37	*G. max*	Landrace	India	Madhya Pradesh	1972	N-9	48315	X
USB-139	PI378658	16.86	*G. max*	Landrace	Ukraine	Dnipropertrovsk	1973	Dnepropetrovsk 12	48190	0
USB-140	PI378663	24.86	*G. max*	Landrace	Russia	ND	1973	Habarovskaja II	48393	I
USB-141	PI378680E	28.13	*G. max*	Landrace	Russia	ND	1973	VNIIMK 9186	48387	I
USB-143	PI379618	15.86	*G. max*	Landrace	Taiwan	ND	1973	TC 1	48254	V
USB-144	PI391577	23.36	*G. max*	Landrace	China	Jilin	1974	Ch'a ye sheng tou	48396	II
USB-145	PI391583	18.35	*G. max*	Landrace	China	Jilin	1974	Jilin No. 10	48264	II

USB-146	PI398633	17.50	*G. max*	Landrace	Korea	Chungcheongbuk-do	1974	KAS 390-17-2	48289	V
USB-147	PI398965	19.87	*G. max*	Landrace	Korea	ND	ND	ND	48313	IV
USB-148	PI404187	30.97	*G. max*	Landrace	China	ND	1975	Suj nii hun mao ju	48271	II
USB-149	PI407701	22.57	*G. max*	Landrace	China	ND	ND	ND	48242	I
USB-150	PI407708A	18.13	*G. max*	Landrace	China	Heilongjiang	1975	Feng shou No. 10	48235	0
USB-151	PI407742	15.79	*G. max*	Landrace	China	Shanxi	1976	16	48266	V
USB-152	PI416751	22.92	*G. max*	Landrace	Japan	Tohoku	1974	A-B(D)	48269	I
USB-153	PI416838	13.96	*G. max*	Landrace	Japan	ND	ND	ND	48211	V
USB-154	PI417215	16.51	*G. max*	Landrace	Japan	Kyushu	1974	Ooita Aki Daizu 2	48283	VIII
USB-155	PI417242	17.81	*G. max*	Landrace	China	ND	1974	Pekin dai seitou	48278	II
USB-156	PI417345B	17.97	*G. max*	Landrace	China	ND	1974	Shou outou	48336	IV
USB-157	PI417381	16.70	*G. max*	Landrace	Japan	Hokkaido	ND	ND	48231	0
USB-158	PI417479	21.49	*G. max*	Landrace	Japan	Tohoku	1974	Yougetsu	48367	IV
USB-159	PI417500	17.76	*G. max*	Landrace	Brazil	ND	1974	Escura A	48246	VIII
USB-160	PI417529	16.49	*G. max*	Landrace	Germany	ND	ND	ND	48229	0
USB-161	PI417581	18.21	*G. max*	Landrace	USA	ND	1974	H-060072	48287	V
USB-162	PI423926	23.41	*G. max*	Landrace	Japan	Nagano	1976	Tousan 72	48214	IV
USB-163	PI424038B	18.34	*G. max*	Landrace	Korea	South	1976	74053	48505	V
USB-166	PI424195A	16.93	*G. max*	Landrace	Hungary	ND	1978	ISZ-3	48227	0
USB-167	PI430595	18.72	*G. max*	Landrace	China	Beijing	1978	58-16	48235	IV
USB-168	PI436684	16.76	*G. max*	Landrace	China	Liaoning	1979	Tie-feng 8	48242	III
USB-169	PI437110A	18.29	*G. max*	Landrace	Russia	Jwish	1979	VIR 244	48338	III
USB-170	PI437112A	18.37	*G. max*	Landrace	Russia	Jwish	1979	VIR 249	48304	II
USB-171	PI437127A	22.03	*G. max*	Landrace	Georgia	ND	1979	Imeretinscaja	48269	IV
USB-173	PI437160	26.44	*G. max*	Landrace	Russia	ND	1979	Krasnodarscaja 13	48270	I
USB-174	PI437165A	16.24	*G. max*	Landrace	Russia	Krasnodar	1979	Toncostebel'naja 27	48234	I
USB-175	PI437240	16.05	*G. max*	Landrace	Moldova	ND	1979	CSchi 1069	48349	0
USB-177	PI437376A	16.35	*G. max*	Landrace	Russia	Primorye	1979	Ussurijscaja 308	48215	I
USB-178	PI437485	23.29	*G. max*	Landrace	Russia	Primorye	1979	VIR 1048	48317	II
USB-179	PI437500A	21.04	*G. max*	Landrace	Russia	Primorye	1979	VIR 3810	48288	I
USB-180	PI437505	21.45	*G. max*	Landrace	Russia	Primorye	1979	VIR 3853	48379	II

USB-182	PI437662	18.12	*G. max*	Landrace	China	ND	1979	Gun'-tszu-lin' 658	48462	II
USB-183	PI437685D	24.94	*G. max*	Landrace	China	ND	1979	Phun-zhun	48284	III
USB-184	PI437695A	33.45	*G. max*	Landrace	China	ND	1979	S-185	48463	I
USB-185	PI437776	14.06	*G. max*	Landrace	China	ND	1979	VIR 1302	48240	III
USB-186	PI437788A	15.08	*G. max*	Landrace	China	ND	1979	VIR 3018	48300	II
USB-187	PI437793	15.08	*G. max*	Landrace	China	ND	1979	VIR 3024	48296	II
USB-188	PI437814A	16.06	*G. max*	Landrace	China	ND	1979	An'da	48339	II
USB-189	PI437838	16.28	*G. max*	Landrace	Russia	ND	1979	DV-254	48212	II
USB-191	PI437956B	22.98	*G. max*	Landrace	China	Northeast	1979	VIR 1390	48420	II
USB-192	PI437991B	19.44	*G. max*	Landrace	China	Northeast	1979	VIR 1657	48328	0
USB-193	PI438019B	17.77	*G. max*	Landrace	China	Northeast	1979	VIR 1883	48308	II
USB-195	PI438083	15.39	*G. max*	Landrace	China	Northeast	1979	VIR 2506	48229	II
USB-196	PI438112B	16.26	*G. max*	Landrace	China	Northeast	1979	VIR 2623	48248	III
USB-198	PI438230A	17.94	*G. max*	Landrace	China	Northeast	1979	VIR 4521	48298	I
USB-199	PI438239B	15.49	*G. max*	Landrace	China	Northeast	1979	VIR 4536	48269	I
USB-201	PI438309	20.37	*G. max*	Landrace	China	ND	1979	VIR 3017	48365	I
USB-202	PI438323	16.23	*G. max*	Landrace	France	ND	1979	Grignon 53-F-3	48192	I
USB-203	PI438336	17.25	*G. max*	Landrace	Algeria	ND	1979	Sao 208	48211	0
USB-204	PI438347	18.40	*G. max*	Landrace	Australia	ND	1979	35S.277	48286	VII
USB-206	PI438496B	16.01	*G. max*	Landrace	USA	ND	ND	ND	48256	III
USB-207	PI438496C	17.06	*G. max*	Landrace	China	ND	1979	VIR 195	48246	IV
USB-208	PI438500	17.04	*G. max*	Landrace	USA	ND	1979	Virginia	48288	III
USB-209	PI445824A	18.15	*G. max*	Landrace	Germany	ND	1925	Wolfsthale	48306	0
USB-210	PI458505	18.15	*G. max*	Landrace	China	Liaoning	1981	Da Bai mei	48276	II
USB-212	PI458510	17.04	*G. max*	Landrace	China	Liaoning	1981	Ji Ti No. 1	48251	III
USB-213	PI464896	16.76	*G. max*	Landrace	China	Jilin	1981	Jou Nong No. 5	48261	I
USB-214	PI458912	19.57	*G. max*	Landrace	China	ND	ND	ND	48230	IV
USB-215	PI464923	19.43	*G. max*	Landrace	China	Liaoning	1981	Tie Fen 16	48294	I
USB-216	PI467347	15.81	*G. max*	Landrace	China	Jilin	1982	Zi-hua-cuo-zi	48222	II
USB-217	PI468408B	16.32	*G. max*	Landrace	China	Jilin	1982	Qi Huang No. 1	48230	III
USB-218	PI475820	18.14	*G. max*	Landrace	China	Xinjiang	1982	ND	48288	II
USB-220	PI476352B	18.34	*G. max*	Landrace	Kyrgyzst	ND	1981	VIR 7081	48304	II
USB-221	PI479735	14.54	*G. max*	Landrace	China	Jilin	1983	Silihuang	48222	III
USB-222	PI490766	15.50	*G. max*	Landrace	China	Hebei	1984	Dawudou	48267	III

USB-223	PI495020	18.84	*G. max*	Landrace	China	Beijing	1985	Xu dou 2	48323	IV
USB-224	PI497953	14.55	*G. max*	Landrace	India	Bihar	1980	I.C. 192	48284	X
USB-226	PI497967	19.25	*G. max*	Landrace	India	Jammu	1980	PLSO 96	48368	VII
USB-227	PI504288	15.21	*G. max*	Landrace	Japan	Iwate	1986	S	48421	V
USB-228	PI506933	16.39	*G. max*	Landrace	Japan	ND	ND	ND	48282	IV
USB-229	PI506942	15.71	*G. max*	Landrace	Japan	Tohoku	1987	Koushu rei 235	48252	II
USB-230	PI507017	34.97	*G. max*	Landrace	Japan	ND	ND	ND	48441	VII
USB-232	PI507088	19.64	*G. max*	Landrace	Japan	Kanto	1987	Nattou Kotsubu	48250	VI
USB-233	PI507293B	14.81	*G. max*	Landrace	Japan	ND	ND	ND	48171	III
USB-234	PI507458	15.15	*G. max*	Landrace	Japan	Kanto	1987	Tousan kei BL 521	48227	IV
USB-235	PI507467	19.65	*G. max*	Landrace	Japan	Kanto	1987	Tousan kei F 764	48215	IV
USB-236	PI507471	16.73	*G. max*	Landrace	Japan	Kanto	1987	Tousan kei na 16	48290	III
USB-238	PI507480	17.00	*G. max*	Landrace	Japan	Kanto	1987	Tousan kei YL 24	48164	IV
USB-240	PI514671	15.53	*G. max*	Landrace	China	Heilongjiang	1987	Feng shou No. 7	48231	0
USB-241	PI518727	17.55	*G. max*	Landrace	China	Guangdong	1988	Ju huang	48277	VI
USB-242	PI532463B	15.91	*G. max*	Landrace	China	Hebei	1989	He bei No. 1	48248	III
USB-245	PI538386A	16.04	*G. max*	Landrace	China	Hebei	1990	1886	48339	III
USB-246	PI540552	14.04	*G. max*	Modern cultivar	USA	Ohio	1987	Hoyt	48157	II
USB-248	PI542972	16.81	*G. max*	Modern cultivar	USA	Illinois	1990	H7190	48130	VII
USB-249	PI548162	16.47	*G. max*	Modern cultivar	USA	Illinois	1954	T48	48258	IV
USB-250	PI548169	14.62	*G. max*	Landrace	USA	Illinois	1954	T117	48230	IV
USB-251	PI548178	28.81	*G. max*	Landrace	USA	Illinois	1954	T145	48308	III
USB-252	PI548198	15.92	*G. max*	Landrace	USA	Illinois	1957	T209	48310	III
USB-253	PI548200	17.43	*G. max*	Landrace	USA	Illinois	1957	T211H	48282	IV
USB-254	PI548256	17.95	*G. max*	Modern cultivar	USA	Mississippi	1985	T279	48206	VII
USB-255	PI548336	15.23	*G. max*	Old cultivar	Russia	ND	1952	Habaro	48281	I
USB-256	PI548356	16.87	*G. max*	Old cultivar	Korea	Phyeongyang	1955	Kanro	48231	II
USB-257	PI548359	17.50	*G. max*	Old cultivar	China	Beijing	1950	Kingwa	48271	IV
USB-258	PI548383	15.79	*G. max*	Old cultivar	China	Heilongjiang	1915	Mansoy	48165	III
USB-259	PI548400	16.04	*G. max*	Old cultivar	China	Heilongjiang	1949	Patoka	48271	IV
USB-260	PI548411	12.63	*G. max*	Old cultivar	China	ND	1949	Seneca	48152	II

USB-261	PI548427	15.64	*G. max*	Old cultivar	China	Liaoning	1950	Wilson	48257	IV
USB-262	PI548473	18.47	*G. max*	Old cultivar	China	Jiangsu	1991	Monetta	48263	VII
USB-263	PI548474	16.42	*G. max*	Old cultivar	Korea	Hwanghaipukto	1991	Nanda	48239	VIII
USB-264	PI548479	16.65	*G. max*	Old cultivar	Taiwan	ND	1991	Otootan	48216	VIII
USB-265	PI548521	19.42	*G. max*	Modern cultivar	USA	Iowa	1982	BSR 201	48260	II
USB-266	PI548524	17.42	*G. max*	Modern cultivar	USA	Iowa	1979	Weber	48319	I
USB-268	PI548571	17.47	*G. max*	Modern cultivar	Canada	Ontario	1973	Harlon	48212	I
USB-269	PI548572	17.37	*G. max*	Modern cultivar	Canada	Ontario	1950	Harly	48111	I
USB-270	PI548582	15.33	*G. max*	Modern cultivar	USA	Minnesota	1978	McCall	48178	0
USB-271	PI548619	55.26	*G. max*	Modern cultivar	USA	kansas	1982	Sparks	48377	IV
USB-272	PI548633	22.24	*G. max*	Modern cultivar	USA	Maryland	1970	Wye	48266	IV
USB-274	PI548696	19.37	*G. max*	Modern cultivar	USA	Arkansas	1979	Dortchsoy 67	48273	V
USB-276	PI548978	16.37	*G. max*	Modern cultivar	USA	Texas	1979	Gail	48185	VI
USB-277	PI549017	19.69	*G. max*	Landrace	China	Ningxia	1990	ZYD 3938	48401	IV
USB-278	PI549021A	19.65	*G. max*	Landrace	China	Liaoning	1990	Na hei dou	48455	III
USB-279	PI549028	18.40	*G. max*	Landrace	China	Liaoning	1990	Feng da li	48278	V
USB-281	PI549040	16.09	*G. max*	Landrace	China	Liaoning	1990	ZYD 2704	48441	IV
USB-282	PI556511	14.60	*G. max*	Modern cultivar	USA	ND	1977	A3127	48164	III
USB-283	PI561318A	18.17	*G. max*	Landrace	China	Beijing	1992	Hui nan bai hua xiao hei dou	48236	I
USB-284	PI561371	17.72	*G. max*	Landrace	China	Shanxi	1992	Fen dou 15	48279	IV
USB-285	PI561387	12.08	*G. max*	Landrace	Japan	ND	1992	Kosuzu	48112	V
USB-286	PI561389B	19.90	*G. max*	Landrace	Japan	Natto	1992	Okura Natto	48333	0
USB-287	PI561701	17.45	*G. max*	Modern cultivar	USA	Georgia	1992	G88-20092	48135	IV
USB-290	PI567074B	16.63	*G. max*	Landrace	Indonesia	east java	1993	MARIF 2672	48315	IX
USB-291	PI567173	17.16	*G. max*	Landrace	China	Heilongjiang	1993	Hei he 51	48223	0
USB-293	PI567225	18.24	*G. max*	Landrace	Moldova	ND	1993	Kisinevskaja 90	48276	0
USB-294	PI567226	14.73	*G. max*	Landrace	Russia	ND	1993	Har'kovskaja Zernouk osnaja	48261	0

USB-295	PI567231	14.55	*G. max*	Landrace	China	Sichuan	1990	WJK-PRC-46	48252	VIII
USB-296	PI567238	14.85	*G. max*	Landrace	China	Yunnan	1990	W6 6210	48296	ND
USB-297	PI567626A	16.18	*G. max*	Landrace	China	Henan	ND	ND	48272	IV
USB-298	PI567307	15.63	*G. max*	Landrace	China	Gansu	1993	Hei huang dou	48236	IV
USB-301	PI567346	14.85	*G. max*	Landrace	China	Gansu	1993	Niu mao huang dou	48327	V
USB-302	PI567352A	15.85	*G. max*	Landrace	China	Gansu	1993	Yang yan qing dou	48296	IV
USB-303	PI567353	17.10	*G. max*	Landrace	China	Gansu	ND	ND	48245	IV
USB-304	PI567361	16.48	*G. max*	Landrace	China	Ningxia	1993	Lu fang huang dou	48326	III
USB-306	PI567407	46.81	*G. max*	Landrace	China	Shanxi	1993	Xiao dou	48430	V
USB-307	PI567408	26.16	*G. max*	Landrace	China	Shanxi	1993	Xiao jin huang	48376	V
USB-308	PI567410B	31.74	*G. max*	Landrace	China	Shanxi	1993	Yang huang dou	48419	VII
USB-309	PI567415A	30.53	*G. max*	Landrace	China	Shanxi	1993	Bai da huang dou	48393	IV
USB-310	PI567416	28.70	*G. max*	Landrace	China	Shanxi	1993	Bai dou	48510	IV
USB-311	PI567418A	28.63	*G. max*	Landrace	China	Shanxi	1993	Bai hei dou	48363	II
USB-312	PI567428	32.24	*G. max*	Landrace	China	Shanxi	1993	Bai ji yao	48480	IV
USB-313	PI567435B	30.74	*G. max*	Landrace	China	Shanxi	1993	Hei hei dou	48382	III
USB-314	PI567439	28.23	*G. max*	Landrace	China	Shanxi	1993	Hong jia huang dou	48395	V
USB-315	PI567488A	17.83	*G. max*	Landrace	China	Hebei	1993	Di liu huang dou No. 2	48369	IV
USB-316	PI567489A	33.83	*G. max*	Landrace	China	Hebei	1993	Er da li huang dou	48482	IV
USB-317	PI567525	32.12	*G. max*	Landrace	China	Shandong	1993	Cao qing huang dou	48313	II
USB-318	PI567532	16.40	*G. max*	Landrace	China	Shandong	1993	Dai ye xiao huang dou	48348	IV
USB-319	PI567548	33.28	*G. max*	Landrace	China	Shandong	1993	Hua li hu zi	48611	IV
USB-320	PI567576	18.37	*G. max*	Landrace	China	Shandong	1993	PIng ding huang	48325	III
USB-322	PI567675	18.12	*G. max*	Landrace	China	Henan	1993	Yu cheng xiao tie jiao huang	48351	IV

USB-323	PI567685	17.07	*G. max*	Landrace	China	Henan	1993	Zhong mou tie jiao er cao	48365	IV
USB-324	PI567698A	15.23	*G. max*	Landrace	China	Anhui	1993	Fu yang (17)	48354	IV
USB-325	PI567726	16.23	*G. max*	Landrace	China	Anhui	ND	ND	48303	IV
USB-326	PI567746	17.15	*G. max*	Landrace	China	Jiangsu	1993	Pei xian da bai jiao	48302	IV
USB-327	PI567780B	16.97	*G. max*	Landrace	China	Jiangsu	1993	Tong shan zheng ji dou	48312	IV
USB-328	PI567782	17.73	*G. max*	Modern cultivar	Canada	Ontario	1993	OAC Dorado	48234	I
USB-330	PI574477	17.49	*G. max*	Landrace	China	Shanxi	1993	Fen dou 31	48207	IV
USB-331	PI578375B	18.00	*G. max*	Landrace	China	ND	1994	Aan tu dang di hei dou	48294	I
USB-332	PI578412	18.95	*G. max*	Landrace	China	ND	1994	Gong jiao 6308-1'	48224	II
USB-333	PI578493	18.79	*G. max*	Landrace	China	ND	1994	Huang bao zhu	48293	II
USB-334	PI578499A	15.79	*G. max*	Landrace	China	ND	1994	Lu yue bai	48151	II
USB-335	PI578503	35.04	*G. max*	Landrace	China	ND	1994	Tie jia si li huang	48333	I
USB-336	PI578504	17.54	*G. max*	Landrace	China	ND	1994	Xiang dou No. 3	48317	II
USB-338	PI587588A	17.14	*G. max*	Landrace	China	Jiangsu	1996	Tai xing niu mao huang yi	48395	IV
USB-339	PI587588B	16.33	*G. max*	Landrace	China	Jiangsu	1996	Tai xing niu mao huang yi	48232	V
USB-341	PI587712B	17.14	*G. max*	Landrace	China	Hubei	1996	E dou No. 1	48271	V
USB-342	PI587804	16.51	*G. max*	Landrace	China	Hubei	1994	Jing 789	48281	IV
USB-343	PI587811A	16.10	*G. max*	Landrace	China	Hubei	1994	ZDD005777	48260	VIII
USB-345	PI592937	18.23	*G. max*	Landrace	China	ND	1994	Jin dou 14	48291	IV
USB-346	PI592940	18.36	*G. max*	Landrace	China	ND	1994	Jin dou 17	48307	IV
USB-347	PI592952	15.09	*G. max*	Landrace	China	ND	1994	Zheng 77249	48293	III
USB-348	PI592960	16.45	*G. max*	Landrace	China	Heilongjiang	1995	Dong nong 38	48159	I
USB-351	PI593953	15.48	*G. max*	Landrace	China	ND	1995	Sui nong No. 10	48200	I
USB-353	PI594170B	19.04	*G. max*	Landrace	Japan	Akita	1996	Geden shirazu	48335	I

USB-354	PI594456A	28.51	*G. max*	Landrace	China	Sichuan	1996	Xiao jin huang	48375	III
USB-357	PI594880	15.66	*G. max*	Landrace	China	Yunnan	1996	Song zi dou	48265	V
USB-358	PI594922	16.35	*G. max*	Modern cultivar	USA	North Carolina	1996	Graham	48073	V
USB-361	PI597464	27.04	*G. max*	Landrace	China	Zhejiang	1997	Zhe chun No. 3	48347	II
USB-362	PI597471A	30.74	*G. max*	Landrace	China	Zhejiang	1996	Zheye 29	48595	VII
USB-363	PI597478B	17.69	*G. max*	Modern cultivar	Korea	South	1997	Paldalkong	48196	III
USB-364	PI598358	18.45	*G. max*	Modern cultivar	USA	Tennessee	1997	TN 5-95	48127	V
USB-366	PI602502B	16.25	*G. max*	Landrace	China	ND	1997	Xiong yue xiao huang dou	48209	IV
USB-367	PI602993	14.89	*G. max*	Landrace	China	Jiangsu	1997	PI xian ruan tiao zhi	48211	IV
USB-368	PI603162	15.08	*G. max*	Landrace	Korea	ND	1997	GL 2631 /96	48231	IV
USB-369	PI603290	17.35	*G. max*	Landrace	China	ND	1998	Zao shu 18	48324	I
USB-370	PI603345	17.07	*G. max*	Landrace	China	Jilin	1998	ZDD00403	48162	II
USB-371	PI603389	15.70	*G. max*	Landrace	China	Liaoning	1998	Huang ke	48139	II
USB-372	PI603397	16.28	*G. max*	Landrace	China	Liaoning	1998	Hei qi huang da dou	48274	IV
USB-373	PI603399	16.96	*G. max*	Landrace	China	Liaoning	1998	Xiao bai qi	48239	II
USB-374	PI603463	17.98	*G. max*	Landrace	China	Shandong	1998	Dong jie No. 1	48260	II
USB-375	PI603488	18.04	*G. max*	Landrace	China	Shandong	1998	ZDD19294	48267	III
USB-376	PI603492	16.75	*G. max*	Landrace	China	Shandong	1998	Qi hei dou	48268	IV
USB-377	PI603494	16.68	*G. max*	Landrace	China	Shandong	1998	Hai dou zi	48224	IV
USB-378	PI603495B	18.35	*G. max*	Landrace	China	ND	1998	Hong mi lan dou zi	48286	V
USB-381	PI603526	17.20	*G. max*	Landrace	China	Shanxi	1998	Hei you dou	48304	IV
USB-382	PI603549	18.34	*G. max*	Landrace	China	Shanxi	1998	Mei dou	48811	III
USB-383	PI603555	14.97	*G. max*	Landrace	China	ND	ND	ND	48258	IV
USB-384	PI603556	20.04	*G. max*	Landrace	China	ND	ND	ND	48416	III
USB-385	PI603559	15.02	*G. max*	Landrace	China	ND	ND	ND	48304	IV
USB-386	PI603675	17.03	*G. max*	Landrace	China	Jiangsu	1998	Huai yin gua dou jia	48281	III
USB-387	PI603698J	17.39	*G. max*	Landrace	China	ND	ND	ND	48261	0
USB-388	PI603722	19.64	*G. max*	Landrace	China	Sichuan	1998	Nan chong ba yue huang	48277	VIII

USB-389	PI605765B	17.85	*G. max*	Landrace	China	Zhejiang	ND	ND	48303	II
USB-390	PI606374	18.47	*G. max*	Landrace	Vietnam	ND	1998	Cao bang 8	48328	IV
USB-392	PI612730	19.32	*G. max*	Landrace	China	ND	1996	Zhong huong No. 10	48247	II
USB-393	PI612754	17.83	*G. max*	Modern cultivar	China	ND	1999	ZY 645	48366	I
USB-395	PI628812	20.19	*G. max*	Modern cultivar	Brazil	ND	2001	MG/BR-46 (Conquista)	48040	V
USB-396	PI628913	17.01	*G. max*	Modern cultivar	Brazil	ND	2001	BR-30	48244	VI
USB-398	PI628963	18.63	*G. max*	Modern cultivar	Brazil	ND	2001	La Suprema	48198	VII
USB-399	PI631123	19.90	*G. max*	Modern cultivar	USA	Maryland	2002	TW98-1	48153	V
USB-400	PI632418	17.09	*G. max*	Modern cultivar	USA	Maryland	2003	Tara	48164	V
USB-401	PI632650	16.92	*G. max*	Landrace	Vietnam	ND	2003	DT 22	48239	V
USB-402	PI639528B	16.61	*G. max*	Landrace	Russia	Primorye	ND	ND	48247	II
USB-403	PI639543	18.43	*G. max*	Landrace	Russia	Primorye	2005	VIR 3715	48348	II
USB-404	PI639550E	16.09	*G. max*	Landrace	Moldova	ND	ND	ND	48271	II
USB-405	PI639559B	20.33	*G. max*	Landrace	Ukraine	ND	2005	VYTKA 2	48398	II
USB-407	PI639570	19.58	*G. max*	Landrace	Philippines	ND	ND	ND	48274	V
USB-409	PI322692	56.83	*G. max*	Landrace	Australia	ND	1967	Max C.P1159A8	48500	IX
USB-411	PI567788	42.51	*G. max*	Modern cultivar	USA	Louisiana	1993	Bienville	48347	VIII
USB-412	FC3654-1	18.44	*G. max*	Landrace	China	Manchuria	1920	Imported Yellow	48282	IV
USB-414	FC31571	17.15	*G. max*	Landrace	China	ND	1944	Kirin	48202	III
USB-415	FC32033	19.98	*G. max*	Landrace	ND	ND	1954	Brown Derby No. 1	48375	0
USB-416	PI54873	18.10	*G. max*	Landrace	China	Heilongjiang	1922	No. 196	48348	II
USB-417	PI59845	16.14	*G. max*	Landrace	Japan	Akita	1924	Sohgetsu	48302	VI
USB-418	PI60970	15.49	*G. max*	Landrace	China	ND	1924	Huang dou	48244	IV
USB-419	PI62202-2	21.49	*G. max*	Landrace	China	Hebei	1924	936	48359	IV
USB-421	PI68423	20.66	*G. max*	Landrace	China	Heilongjiang	1926	5823	48289	III
USB-422	PI68436	19.06	*G. max*	Landrace	China	Heilongjiang	1926	17	48295	II
USB-423	PI68523	19.98	*G. max*	Landrace	China	Heilongjiang	1926	170	48331	III
USB-424	PI68679-2	19.41	*G. max*	Landrace	China	Manchuria	1926	298	48333	IV
USB-425	PI68731	17.74	*G. max*	Landrace	China	Manchuria	1926	289	48292	III
USB-426	PI68736	19.12	*G. max*	Landrace	China	Manchuria	1926	254	48364	II

USB-427	PI68765	21.73	*G. max*	Landrace	China	Manchuria	1926	65	48416	II
USB-428	PI68815	22.04	*G. max*	Landrace	China	Heilongjiang	1926	6450-m	48476	II
USB-429	PI70078	24.06	*G. max*	Landrace	China	Jilin	1926	6900	48354	II
USB-430	PI70201	20.70	*G. max*	Landrace	China	Jilin	1926	7548	48321	III
USB-431	PI70208	18.39	*G. max*	Landrace	China	Liaoning	ND	ND	48319	IV
USB-432	PI70242-2	17.60	*G. max*	Landrace	China	ND	ND	ND	48445	IV
USB-433	PI79616	15.10	*G. max*	Landrace	China	Liaoning	ND	ND	48274	III
USB-435	PI79797	20.16	*G. max*	Landrace	ND	ND	ND	ND	48307	III
USB-436	PI79832	15.07	*G. max*	Landrace	China	Heilongjiang	ND	ND	48202	V
USB-437	PI79870-4	14.49	*G. max*	Landrace	China	Heilongjiang	ND	ND	48236	IV
USB-438	PI80466-1	17.11	*G. max*	Landrace	Japan	Hokkaido	ND	ND	48294	III
USB-439	PI80671	17.56	*G. max*	Landrace	Japan	Tokyo	ND	ND	48304	II
USB-440	PI81042-2	24.26	*G. max*	Landrace	Japan	Hokkaido	ND	ND	48300	IV
USB-441	PI82302	20.39	*G. max*	Landrace	Korea	Seoul	ND	ND	48328	III
USB-442	PI83925	20.07	*G. max*	Landrace	Japan	Akita	ND	ND	48349	IV
USB-443	PI84509	17.87	*G. max*	Landrace	ND	ND	ND	ND	48324	III
USB-444	PI84660	13.65	*G. max*	Landrace	Korea	Kyonggi	ND	ND	48323	IV
USB-445	PI84734	20.02	*G. max*	Landrace	Korea	Kyonggi	ND	ND	48400	VI
USB-446	PI86084	21.40	*G. max*	Landrace	Japan	Hokkaido	ND	ND	48353	V
USB-447	PI86982	19.29	*G. max*	Landrace	Korea	Cholla Puk	ND	ND	48525	V
USB-448	PI87076	18.44	*G. max*	Landrace	Korea	Hwanghaipukto	ND	ND	48288	V
USB-449	PI87571	16.76	*G. max*	Landrace	Korea	Phyeongyang	ND	Kokukaku	48333	IV
USB-450	PI87618	22.68	*G. max*	Landrace	Korea	Hamkysongpukto	ND	ND	48341	III
USB-451	PI87634	17.86	*G. max*	Landrace	Japan	Saitama	ND	ND	48285	III
USB-452	PI88306	17.60	*G. max*	Landrace	China	Liaoning	ND	ND	48297	III
USB-453	PI88448	15.75	*G. max*	Landrace	China	ND	ND	ND	48302	III
USB-454	PI88479	23.04	*G. max*	Landrace	China	Jilin	ND	ND	48333	II
USB-455	PI88499	22.59	*G. max*	Landrace	China	Liaoning	ND	ND	48308	IV
USB-456	PI88508	14.98	*G. max*	Landrace	China	Liaoning	ND	ND	48577	II
USB-459	PI90369	20.07	*G. max*	Landrace	China	ND	ND	ND	48536	IV
USB-460	PI90406-1	15.53	*G. max*	Landrace	China	Beijing	ND	ND	48327	IV
USB-462	PI90495N	22.02	*G. max*	Landrace	China	ND	ND	ND	48341	IV
USB-463	PI90499-1	13.62	*G. max*	Landrace	China	Hebei	ND	ND	48259	III
USB-464	PI90577	14.46	*G. max*	Landrace	China	Northeast	ND	ND	48394	VI
USB-465	PI90723	17.36	*G. max*	Landrace	ND	ND	ND	ND	48419	III

USB-466	PI90768	19.63	*G. max*	Landrace	China	Beijing	ND	ND	48383	VI
USB-467	PI91083	16.29	*G. max*	Landrace	Korea	ND	ND	ND	48389	III
USB-468	PI91089	17.84	*G. max*	Landrace	China	Jilin	ND	ND	48292	III
USB-469	PI91120	14.46	*G. max*	Landrace	China	Jilin	ND	ND	48218	II
USB-470	PI91132-3	18.51	*G. max*	Landrace	China	Jilin	ND	ND	48312	IV
USB-472	PI91725	18.42	*G. max*	Landrace	Korea	Phyeongyang	ND	ND	48288	V
USB-473	PI91731-1	16.80	*G. max*	Landrace	China	Jilin	ND	ND	48280	IV
USB-474	PI92601	19.82	*G. max*	Landrace	China	Jilin	ND	ND	48343	VI
USB-475	PI92604	19.77	*G. max*	Landrace	China	Jilin	ND	ND	48279	IV
USB-476	PI92618	17.31	*G. max*	Landrace	China	Jilin	ND	ND	48201	III
USB-477	PI92688-2	18.97	*G. max*	Landrace	China	Jilin	ND	ND	48304	IV
USB-478	PI92718-2	19.18	*G. max*	Landrace	China	Jilin	ND	ND	48314	III
USB-479	PI92728	21.28	*G. max*	Landrace	China	Jilin	ND	ND	48301	III
USB-480	PI93055	23.67	*G. max*	Landrace	China	Zhejiang	ND	ND	48469	IV
USB-481	PI93563	20.32	*G. max*	Landrace	China	Jilin	ND	ND	48352	III
USB-484	PI103079	18.59	*G. max*	Landrace	China	Henan	1933	Shang tsai	48339	V
USB-485	PI123439	17.19	*G. max*	Landrace	Myanmar	ND	1937	No. 1	48334	VII
USB-486	PI123587	16.61	*G. max*	Landrace	China	Hebei	1937	Hopei E602	48336	V
USB-488	PI165674	17.11	*G. max*	Landrace	China	Jiangsu	1948	Liuchow B	48341	VIII
USB-489	PI165929	15.04	*G. max*	Landrace	India	Uttar Pradesh	1948	Bhart	48356	VII
USB-490	PI171438	18.58	*G. max*	Landrace	China	Sichuan	1948	Hwa-an	48422	VII
USB-492	PI180532	16.72	*G. max*	Old cultivar	Germany	ND	1949	Strain No. 1379/47	48374	0
USB-494	PI192870	19.99	*G. max*	Landrace	Indonesia	Jawa	1950	No. 441	48393	VII
USB-496	PI198067	19.96	*G. max*	Old cultivar	Sweden	Malmohus	1951	Ugra Saja	48304	0
USB-497	PI200503	18.09	*G. max*	Landrace	Japan	ND	1952	Miyashiro jun	48328	V
USB-498	PI209331	20.16	*G. max*	Landrace	Japan	Hokkaido	1953	No. 1	48354	III
USB-499	PI219698	19.20	*G. max*	Landrace	Pakistan	Northern Areas	1954	Kulat	48425	VI
USB-500	PI227320	15.93	*G. max*	Landrace	Japan	Hokkaido	1955	Daiichihienuki	48432	IV
USB-502	PI235347	18.73	*G. max*	Landrace	Japan	ND	1956	ND	48288	IV
USB-504	PI253652B	20.92	*G. max*	Landrace	China	ND	1958	No. 3	48275	IV
USB-505	PI253656B	21.96	*G. max*	Landrace	China	ND	1958	No. 7	48406	IV
USB-506	PI257429	22.06	*G. max*	Old cultivar	Germany	Nordrhein-Westfalen	1959	Soja-C.-St. 4/58	48331	0
USB-510	PI290136	15.92	*G. max*	Landrace	France	ND	1963	Noir 1	48279	0

USB-512	PI319527	23.34	*G. max*	Landrace	China	ND	1967	PIn tin hwang	48367	V
USB-514	PI325779	20.97	*G. max*	Landrace	Brazil	Salvador	1968	Lucerna	48322	IX
USB-516	PI341245	21.32	*G. max*	Landrace	Tanzania	ND	1969	Avoyelles	48470	IX
USB-518	PI370057B	21.33	*G. max*	Landrace	Russia	Primorye	1972	VIR 5525	48351	II
USB-520	PI377574	12.62	*G. max*	Landrace	Japan	Iwate	1972	Hakuto	48439	IV
USB-521	PI378670B	19.79	*G. max*	Landrace	Moldova	ND	1973	Kisinevskaja 1	48354	II
USB-522	PI378682C	21.97	*G. max*	Landrace	Japan	Akita	1973	ND	48446	IV
USB-524	PI399058	21.71	*G. max*	Landrace	Korea	Kangwon	1974	Kangwon	48360	V
USB-525	PI404161	11.52	*G. max*	Landrace	Georgia	ND	1975	Mocinabe 7	48161	IV
USB-526	PI404182	22.09	*G. max*	Landrace	China	ND	1975	Sin i tu li rau	48385	III
USB-527	PI404199	15.03	*G. max*	Landrace	China	ND	1975	Sin he do	48377	IV
USB-528	PI407659B	17.95	*G. max*	Landrace	China	ND	ND	ND	48398	III
USB-529	PI407717	15.83	*G. max*	Landrace	China	Jilin	1975	Jin nung No. 5	48302	II
USB-530	PI408088	15.62	*G. max*	Landrace	Korea	Kyongsang Puk	1975	KAERI 633-2	48339	V
USB-531	PI416768	15.84	*G. max*	Landrace	Japan	Nagano	1974	Akasaya (Nagano)	48346	V
USB-533	PI417007	22.67	*G. max*	Landrace	Japan	ND	1974	Karasumame	48423	IV
USB-534	PI417077	15.67	*G. max*	Landrace	Japan	ND	1974	Koushurei 273	48311	III
USB-537	PI417550	23.07	*G. max*	Landrace	Russia	ND	1974	Amuvskaje	48362	0
USB-538	PI424005	16.36	*G. max*	Landrace	Korea	Kyonggi	1976	74002	48446	III
USB-540	PI430596	23.24	*G. max*	Landrace	China	Fujian	1978	Fu pe tou	48440	II
USB-541	PI430598B	21.20	*G. max*	Landrace	China	Fujian	1978	Shuang ching tou	48341	IV
USB-543	PI434974	21.43	*G. max*	Landrace	China	ND	1979	Seminole	48477	IX
USB-544	PI437123	17.48	*G. max*	Landrace	Russia	ND	1979	VIR 4575	48262	I
USB-545	PI437135B	17.47	*G. max*	Landrace	Russia	ND	1979	Amurzejscaja	48303	II
USB-546	PI437138	23.83	*G. max*	Landrace	Russia	Krasnodar	1979	Chabarovscaja 4	48391	0
USB-548	PI437168A	15.32	*G. max*	Landrace	Russia	Krasnodar	1979	VNIISC-2	48362	I
USB-549	PI437211B	14.70	*G. max*	Landrace	Moldova	ND	1979	CSchi 44	48384	0
USB-550	PI437287	17.96	*G. max*	Landrace	Moldova	ND	1979	VIR 4931	48362	0
USB-551	PI437296	16.15	*G. max*	Landrace	Moldova	ND	1979	VIR 4944	48340	0
USB-553	PI437321	16.09	*G. max*	Landrace	Russia	Primorye	1979	Dunganscaja 462	48302	III
USB-554	PI437326	20.36	*G. max*	Landrace	Russia	Primorye	1979	DV-184	48346	II
USB-555	PI437347	16.38	*G. max*	Landrace	Russia	Primorye	1979	Primorscaja 8	48436	II

USB-556	PI437366	17.39	*G. max*	Landrace	Russia	Primorye	1979	Ussurijscaja 155	48302	I
USB-558	PI437427A	17.26	*G. max*	Landrace	Russia	Primorye	1979	Ussurijscaja 547	48410	II
USB-559	PI437431	16.53	*G. max*	Landrace	Russia	Primorye	1979	Ussurijscaja 559	48267	II
USB-560	PI437476	17.77	*G. max*	Landrace	Russia	Primorye	1979	VIR 381	48386	III
USB-561	PI437477B	16.23	*G. max*	Landrace	Russia	Primorye	1979	VIR 382	48345	II
USB-562	PI437487	13.96	*G. max*	Landrace	Russia	Primorye	1979	VIR 1069	48291	III
USB-563	PI437513	15.85	*G. max*	Landrace	Russia	Primorye	1979	VIR 4210	48261	II
USB-564	PI437562	17.40	*G. max*	Landrace	China	ND	1979	Bej-sjan'-ni-dou	48386	VIII
USB-565	PI437578	21.26	*G. max*	Landrace	China	ND	1979	Chuan-da-dou	48261	III
USB-566	PI437594A	20.24	*G. max*	Landrace	China	ND	1979	Dun-haj-sy-tszjao	48626	I
USB-567	PI437609B	16.52	*G. max*	Landrace	China	ND	1979	DV-2780	48289	II
USB-568	PI437615A	15.37	*G. max*	Landrace	China	ND	1979	DV-2788	48436	0
USB-569	PI437634	16.49	*G. max*	Landrace	China	ND	1979	DV-2815	48327	I
USB-570	PI437640A	16.23	*G. max*	Landrace	China	ND	1979	DV-2821	48385	I
USB-571	PI437721B	16.53	*G. max*	Landrace	China	ND	1980	Sy-jue-tsin-mao-do	48395	I
USB-573	PI437781	16.03	*G. max*	Landrace	China	ND	1979	VIR 1330	48359	I
USB-574	PI437798	15.69	*G. max*	Landrace	China	ND	1979	VIR 5505	48343	IV
USB-575	PI437815	15.85	*G. max*	Landrace	China	Northeast	1979	An,dunscaja	48287	I
USB-576	PI437875A	15.35	*G. max*	Landrace	China	ND	1979	Elita 691	48290	I
USB-577	PI437995A	16.74	*G. max*	Landrace	China	Northeast	1979	VIR 1673	48334	0
USB-578	PI437999	16.96	*G. max*	Landrace	China	Northeast	1979	VIR 1711	48320	0
USB-579	PI438011	14.84	*G. max*	Landrace	China	Northeast	1979	VIR 1813	48282	II
USB-580	PI438047	15.48	*G. max*	Landrace	China	Northeast	1979	VIR 2303	48334	III
USB-581	PI438120	15.02	*G. max*	Landrace	China	Northeast	1979	VIR 2785	48324	I
USB-582	PI438195	16.52	*G. max*	Landrace	China	Northeast	1979	VIR 4476	48331	I
USB-583	PI438267	14.14	*G. max*	Landrace	China	Northeast	1979	VIR 5041	48312	0
USB-584	PI438310	17.77	*G. max*	Landrace	Korea	ND	1979	DV-2342	48312	III
USB-585	PI438312	17.62	*G. max*	Landrace	Algeria	ND	1979	Blaen Small	48494	III
USB-586	PI438329	14.07	*G. max*	Landrace	Algeria	ND	1979	Reaz 20/46-230	48266	II
USB-587	PI438358B	15.71	*G. max*	Landrace	Bulgaria	ND	1979	VU-5819	48343	0
USB-589	PI438450	22.11	*G. max*	Landrace	Poland	ND	1979	K.P. 3025	48397	II
USB-590	PI438492	19.51	*G. max*	Landrace	USA	ND	1979	Elton	48308	I

USB-591	PI438504B	22.14	*G. max*	Landrace	USA	ND	1979	VIR 24a	48386	II
USB-592	PI438509A	23.42	*G. max*	Landrace	USA	ND	1979	VIR 202	48432	I
USB-593	PI464913	18.65	*G. max*	Landrace	China	Liaoning	1981	Dan Dou 2	48302	IV
USB-594	PI466749A	21.22	*G. max*	Landrace	China	Shanghai	1982	Wu Yui Huo	48411	II
USB-595	PI467313	21.68	*G. max*	Landrace	China	Jilin	1982	Da-hei-qi	48356	0
USB-596	PI468919	19.84	*G. max*	Landrace	China	Liaoning	1982	ND	48705	III
USB-597	PI475812B	17.85	*G. max*	Landrace	China	Xinjiang	1982	ND	48374	III
USB-599	PI476344	16.24	*G. max*	Landrace	Uzbekistan	ND	1981	Uzbekskaja-2	48268	II
USB-602	PI476939	17.43	*G. max*	Landrace	Vietnam	ND	1982	Den Ha Bac	48344	IV
USB-603	PI483253	16.06	*G. max*	Landrace	Brazil	ND	1984	TroPIcal	48349	IX
USB-604	PI504495	16.38	*G. max*	Landrace	Taiwan	ND	1986	Kaohsiung No. 2	48401	V
USB-607	PI507678	17.63	*G. max*	Old cultivar	Russia	ND	1987	Fakel	48263	I
USB-608	PI508083	15.01	*G. max*	Modern cultivar	USA	Minnesota	1987	Dassel	48267	0
USB-609	PI508269	17.11	*G. max*	Modern cultivar	USA	Virginia	1987	Stafford	48195	IV
USB-611	PI518711	15.68	*G. max*	Landrace	China	Jilin	1988	Jilin 21	48319	II
USB-612	PI518759	15.43	*G. max*	Landrace	Taiwan	ND	1988	Kaohsiung 10	48274	VI
USB-613	PI525454	13.76	*G. max*	Modern cultivar	USA	Indiana	1988	Spencer	48252	IV
USB-614	PI532454	13.88	*G. max*	Landrace	China	Jiangsu	1989	Tai xing hei dou	48315	0
USB-615	PI533654	18.19	*G. max*	Modern cultivar	USA	Illinois	1988	LN83-2356	48285	IV
USB-616	PI534648	14.93	*G. max*	Modern cultivar	USA	Ohio	1988	GR8936	48304	III
USB-617	PI539865	13.19	*G. max*	Modern cultivar	USA	Iowa	1990	HP204	48284	I
USB-618	PI539936	13.11	*G. max*	Modern cultivar	USA	ND	1990	A4715	48203	IV
USB-619	PI540555	16.66	*G. max*	Modern cultivar	USA	ND	ND	ND	48279	IV
USB-620	PI543794	17.43	*G. max*	Modern cultivar	USA	Missouri	1990	Delsoy 4900	48163	IV
USB-622	PI547831	18.80	*G. soja*	Wild soybean	USA	Illinois	1989	L83-0215	48642	II
USB-623	PI548158	19.62	*G. max*	Modern cultivar	USA	Illinois	1954	T16	48315	IV
USB-628	PI548414	14.95	*G. max*	Old cultivar	Japan	Hokkaido	1957	Sioux	48406	0
USB-629	PI548440	14.56	*G. max*	Old cultivar	USA	Arizona	1942	Armredo	48333	VI
USB-630	PI548464	15.07	*G. max*	Old cultivar	China	Tianjin	1906	Lexington	48381	V

USB-632	PI548472	15.46	*G. max*	Old cultivar	China	Jiangsu	1991	Missoy	48427	VII
USB-633	PI548491	13.48	*G. max*	Old cultivar	China	Shanghai	1991	Tarheel Black	48310	VII
USB-634	PI548497	14.68	*G. max*	Old cultivar	USA	South Carolina	1991	Yelredo	48373	VIII
USB-635	PI548528	15.91	*G. max*	Modern cultivar	USA	Indiana	1966	Protana	48433	II
USB-637	PI548547	14.79	*G. max*	Modern cultivar	USA	Indiana	1967	Cutler	48388	IV
USB-640	PI548568	14.81	*G. max*	Modern cultivar	USA	Wisconsin	1955	Grant	48358	0
USB-642	PI549023B	17.59	*G. max*	Landrace	China	Liaoning	1990	Na xiao lu dou	48335	V
USB-646	PI556637	21.12	*G. max*	Modern cultivar	USA	ND	1991	A1937	48258	I
USB-650	PI561321	22.09	*G. max*	Landrace	China	Beijing	1992	Hui nan zi hua xiao hei dou	48391	III
USB-654	PI566998A	21.29	*G. max*	Landrace	Indonesia	ND	1993	MARIF 2547	48327	VIII
USB-655	PI567056A	18.07	*G. max*	Landrace	Indonesia	ND	1993	MARIF 2649	48501	VIII
USB-656	PI567060A	13.67	*G. max*	Landrace	Indonesia	ND	1993	MARIF 2656	48300	V
USB-658	PI567170A	14.87	*G. max*	Landrace	China	Jilin	1993	He qi PIng ding xiang	48242	II
USB-663	PI567265	15.44	*G. max*	Landrace	China	Fujian	1993	NC 9180	48234	IV
USB-664	PI567272A	15.75	*G. max*	Landrace	Taiwan	ND	1993	AV 215	48286	IV
USB-666	PI567331	17.43	*G. max*	Landrace	China	Gansu	1993	Huang PI xiao huang dou	48379	IV
USB-669	PI567366A	18.97	*G. max*	Landrace	China	Ningxia	1993	Wu tong shu huang dou	48447	III
USB-670	PI567370A	17.36	*G. max*	Landrace	China	Ningxia	1993	Xu wang huang da dou	48330	IV
USB-672	PI567381B	17.74	*G. max*	Landrace	China	Shanxi	1993	Bai ke huang	48296	V
USB-673	PI567390	20.22	*G. max*	Landrace	China	Shanxi	1993	Ji dan PI bai dou	48337	V
USB-674	PI567392	16.99	*G. max*	Landrace	China	Shanxi	1993	Jing yang hong da dou	48343	V
USB-675	PI567394B	18.04	*G. max*	Landrace	China	Shanxi	1993	Jiu yue han	48376	VI
USB-676	PI567395	19.33	*G. max*	Landrace	China	Shanxi	ND	ND	48417	IV
USB-677	PI567396B	15.65	*G. max*	Landrace	China	Shanxi	1993	Lao shu PI	48325	IV
USB-678	PI567398	18.53	*G. max*	Landrace	China	Shanxi	1993	Lu lan dou	48368	IV

USB-679	PI567404A	24.57	*G. max*	Landrace	China	Shanxi	1993	Wang shan hou	48323	III
USB-680	PI567405	20.75	*G. max*	Landrace	China	Shanxi	1993	Wei zi dou	48378	VI
USB-682	PI567417B	12.09	*G. max*	Landrace	China	Shanxi	1993	Bai gun dou	48245	I
USB-684	PI567458	11.71	*G. max*	Landrace	China	Shanxi	1993	Ji li huang dou	48306	IV
USB-687	PI567503	9.69	*G. max*	Landrace	China	Hebei	1993	Niu mao huang dou	48184	IV
USB-688	PI567541B	21.27	*G. max*	Landrace	China	Shandong	1993	Gun li huang	48368	III
USB-689	PI567552	17.48	*G. max*	Landrace	China	Shandong	1993	Huang si li	48421	IV
USB-690	PI567556	22.26	*G. max*	Landrace	China	Shandong	1993	Kao shan hong	48502	IV
USB-691	PI567559	18.62	*G. max*	Landrace	China	Shandong	1993	Liu yue xian	48365	II
USB-694	PI567582A	19.79	*G. max*	Landrace	China	Shandong	1993	Qi si wa	48391	IV
USB-696	PI567593B	16.56	*G. max*	Landrace	China	Shandong	1993	Xiao bai dou	48320	IV
USB-697	PI567599	21.18	*G. max*	Landrace	China	Shandong	1993	Xiao PIng ding huang	48382	III
USB-699	PI567638	17.43	*G. max*	Landrace	China	Henan	1993	Min quan wan dou yuan	48452	IV
USB-700	PI567648C	18.45	*G. max*	Landrace	China	Henan	1993	Qin yang hui jiao cao bai dou	48380	IV
USB-701	PI567721	18.14	*G. max*	Landrace	China	Anhui	1993	Fu yang (45)	48362	IV
USB-703	PI567772	21.77	*G. max*	Landrace	China	Jiangsu	1993	Tong shan hong mao you	48388	IV
USB-705	PI574478C	14.97	*G. max*	Landrace	China	Heilongjiang	1993	Hai jiao zhao	48247	II
USB-706	PI578316C	16.22	*G. max*	Landrace	Nepal	ND	1994	ND	48384	VIII
USB-707	PI578329C	25.43	*G. max*	Modern cultivar	Argentina	ND	1994	OFPEC Cordobesa	48298	VI
USB-708	PI578330	19.00	*G. max*	Modern cultivar	Argentina	ND	1994	OFPEC Nortena	48316	VIII
USB-710	PI578490	17.94	*G. max*	Landrace	China	ND	1994	He nan zao feng No. 1	48347	IV
USB-711	PI583366	21.44	*G. max*	Modern cultivar	USA	Virginia	1994	Chesapeake	48216	IV

USB-712	PI587560A	20.21	*G. max*	Landrace	China	Jiangsu	1995	Dan tu ba yue bai jia	48458	VII
USB-713	PI587575A	17.94	*G. max*	Landrace	China	Jiangsu	1994	Sha zhou jie jie si	48353	IV
USB-715	PI587582	18.63	*G. max*	Landrace	China	Jiangsu	1994	Jiang pu huang da dou jia	48328	VI
USB-717	PI587668A	17.11	*G. max*	Landrace	China	Anhui	1994	Hui mei dou	48372	VI
USB-718	PI587788B	17.95	*G. max*	Landrace	China	Jiangsu	1994	Nan zhang hei huang dou	48379	VI
USB-719	PI587794	21.42	*G. max*	Landrace	China	Hubei	1994	Wu ming 18 jia	48374	VI
USB-728	PI587963	18.42	*G. max*	Landrace	China	Hunan	1994	Yao dou	48354	X
USB-729	PI588015B	17.06	*G. max*	Landrace	China	Sichuan	1994	Bai mao dou	48334	IV
USB-730	PI588026C	17.74	*G. max*	Landrace	China	Sichuan	1994	Luo xi	48327	IV
USB-732	PI592919	18.06	*G. max*	Landrace	China	ND	1994	ZDD 17688	48352	I
USB-733	PI592928	16.95	*G. max*	Landrace	China	ND	1994	Gong dou No. 4	48323	II
USB-734	PI592932	18.88	*G. max*	Landrace	China	ND	1994	Zao shu No. 9	48343	II
USB-735	PI592936	21.17	*G. max*	Landrace	China	ND	1994	ZDD 18632	48291	II
USB-737	PI594409B	16.91	*G. max*	Landrace	China	Anhui	1996	86-8-39	48317	III
USB-740	PI594467	16.19	*G. max*	Landrace	China	Sichuan	1996	Zi PI dou	48331	III
USB-741	PI594470C	17.90	*G. max*	Landrace	China	Sichuan	1996	Zao dou zi	48376	VIII
USB-742	PI594487	18.14	*G. max*	Landrace	China	ND	ND	ND	48486	VIII
USB-743	PI594497	19.65	*G. max*	Landrace	China	Sichuan	1996	Za se huang dou No. 3	48396	VIII
USB-745	PI594660A	18.38	*G. max*	Landrace	China	Guizhou	1996	Liu yue dou No. 1	48375	V
USB-748	PI594753A	19.71	*G. max*	Landrace	China	Guangxi	1996	Hei qi da dou	48437	VII
USB-749	PI594757	18.21	*G. max*	Landrace	China	Guangxi	ND	ND	48401	IX
USB-750	PI594769	19.25	*G. max*	Landrace	China	Guangxi	1996	Luo wei qing dou	48407	IX
USB-751	PI594805A	16.91	*G. max*	Landrace	China	Yunnan	1996	Huang PI dou	48363	VII
USB-752	PI594810B	14.91	*G. max*	Landrace	China	Yunnan	1996	Xiao huang mao dou	48434	VI
USB-753	PI594822	19.23	*G. max*	Landrace	China	Yunnan	1996	Xi huang dou	48436	IX

USB-754	PI594830	20.37	*G. max*	Landrace	China	Yunnan	1996	Lu huang dou	48490	X
USB-755	PI594834A	19.09	*G. max*	Landrace	China	Yunnan	1996	Wu yue bai dou	48360	VII
USB-756	PI594839A	20.43	*G. max*	Landrace	China	Yunnan	1996	Huang dou	48504	VIII
USB-758	PI594883	16.80	*G. max*	Landrace	China	Yunnan	1996	De hong xuan No. 8	48328	IV
USB-759	PI597384	19.36	*G. max*	Modern cultivar	USA	Illinois	1997	LN89-3615	48277	IV
USB-761	PI597402	23.57	*G. max*	Landrace	Russia	Krasnodar	1996	Runo	48338	0
USB-762	PI602492	20.22	*G. max*	Landrace	China	Liaoning	1997	Liao dou 12	48656	III
USB-763	PI603174A	17.42	*G. max*	Landrace	Korea	ND	1997	ND	48377	IV
USB-765	PI603308A	17.36	*G. max*	Landrace	China	ND	1998	Niu xin hong	48377	I
USB-768	PI603356	15.71	*G. max*	Landrace	China	Jilin	1998	ZDD00475	48312	I
USB-769	PI603401	17.98	*G. max*	Landrace	China	Liaoning	1998	ZDD01149	48278	IV
USB-771	PI603408	19.55	*G. max*	Landrace	China	Liaoning	1998	ZDD01288	48410	I
USB-772	PI603412A	17.42	*G. max*	Landrace	China	ND	1998	ZDD001460	48463	II
USB-773	PI603421B	18.69	*G. max*	Landrace	China	ND	1998	ZDD001502	48481	III
USB-776	PI603446	16.71	*G. max*	Landrace	China	Neimongoi	1998	ZDD18377	48335	II
USB-777	PI603451A	16.51	*G. max*	Landrace	China	ND	1998	ZDD002757	48320	III
USB-778	PI603457B	18.15	*G. max*	Landrace	China	ND	1998	Mi lan dou zi	48331	IV
USB-779	PI603477A	20.56	*G. max*	Landrace	China	ND	1998	Jiang nan qing	48356	III
USB-780	PI603490	13.66	*G. max*	Landrace	China	Shandong	1998	Hei you dou	48298	IV
USB-781	PI603496B	15.76	*G. max*	Landrace	China	ND	1998	Xiao hong dou	48329	IV
USB-782	PI603502A	15.64	*G. max*	Landrace	China	ND	1998	ZDD010273	48669	III
USB-783	PI603505	15.43	*G. max*	Landrace	China	ND	1998	ZDD10371	48465	IV
USB-784	PI603510	13.82	*G. max*	Landrace	China	Shanxi	1998	Xiao li bai	48393	V
USB-786	PI603523	22.76	*G. max*	Landrace	China	Shanxi	1998	ZDD10686	48383	V
USB-787	PI603532	16.87	*G. max*	Landrace	China	Shanxi	1998	Hong li huang dou	48380	VI
USB-788	PI603534B	19.19	*G. max*	Landrace	China	ND	2000	Da niu mao huang	48384	VIII
USB-792	PI603554B	18.40	*G. max*	Landrace	China	ND	ND	ND	48416	IV
USB-793	PI603557	14.70	*G. max*	Landrace	China	ND	ND	ND	48404	IV
USB-794	PI603574	16.76	*G. max*	Landrace	China	Shanxi	1998	ZDD08897	48397	IV
USB-795	PI603583	22.25	*G. max*	Landrace	China	Shanxi	1998	ZDD09057	48438	IV
USB-798	PI603687A	17.06	*G. max*	Landrace	China	ND	ND	ND	48320	IV

USB-799	PI603696	16.55	*G. max*	Landrace	China	Jiangsu	1998	Shu yang qiu dou jia	48432	VI
USB-800	PI603740B	20.11	*G. max*	Landrace	China	Sichuan	1998	Ma you bao No. 2	48421	IX
USB-801	PI603910C	16.16	*G. max*	Landrace	Korea	ND	1998	Cin	48370	V
USB-802	PI603911B	16.50	*G. max*	Landrace	Korea	ND	1998	Jijori	48331	III
USB-803	PI605839A	19.91	*G. max*	Landrace	Vietnam	Hagiang	1998	Xam si man	48353	IV
USB-805	PI619615	16.84	*G. max*	Modern cultivar	USA	North Carolina	2001	N6201	48306	VI
USB-806	PI628834	18.30	*G. max*	Modern cultivar	Brazil	ND	2001	FT-16	48347	VIII
USB-807	PI628867	19.49	*G. max*	Modern cultivar	Brazil	ND	2001	Ocepar-3 (Primavera)	48318	VI
USB-812	PI632654	19.41	*G. max*	Landrace	Vietnam	ND	2003	VG 4763	48565	V
USB-814	PI632943B	21.24	*G. max*	Landrace	Vietnam	Dong Nai	2001	Dau nanh	48573	VII
USB-818	PI653874B	25.28	*G. max*	Landrace	Vietnam	ND	2008	Viet. Donor No. 29	48385	II
USB-819	PI656647	18.96	*G. max*	Modern cultivar	USA	Mississippi	2009	DS4-SCN05	48289	IV
