# Supplementary material for: Machine learning models outperform deep learning models, provide interpretation and facilitate feature selection for soybean trait prediction
Source: BMC Plant Biol. 2022 Apr 8;22:180. doi: 10.1186/s12870-022-03559-z (PMC8991976; doi:10.1186/s12870-022-03559-z)
Supplement: Supplementary file 1 — Additional file 1: Supplementary Figure 1. P-value of each SNPs association for a) flower colour b) seed coat colour c) pod colour in the soybean VCF. SNPs coloured red have been determined as significantly associated for the given trait as they have a p-value less than the -log10(8) significance threshold for this GWAS. Supplementary Figure 2. Graphs ranking the top 20 most input SNPs by gain as identified by XGBoost models for trait predictions for traits with regions of importance identified from XGBoost. Blue bars are region of importance, whereas other colours represent collections of important SNPs on the same chromosome. Black bars represent left over SNPs with no relation to other SNPs in the ranking. SNP rankings for genome wide SNP input for A) flower colour B) seed coat colour C) pubescence density D) seed weight. Supplementary Figure 3. Top 20 ranked SNPs for XGBoost Seed Oil Prediction. Supplementary Figure 4. Top 20 ranked SNPs for XGBoost Pod Colour Prediction. Supplementary Figure 5. Top 20 ranked SNPs for XGBoost Seed Protein Prediction. Supplementary Table 1. Targeted Regions of SNPs for Reduced Input Models. Supplementary Table 2. List of soybean germplasm in the pangenome with the sequence coverage. (ND, not defined). Supplementary Table 3. Trait Data Types. [file 12870_2022_3559_MOESM1_ESM.zip › Supplementary Figures.docx]

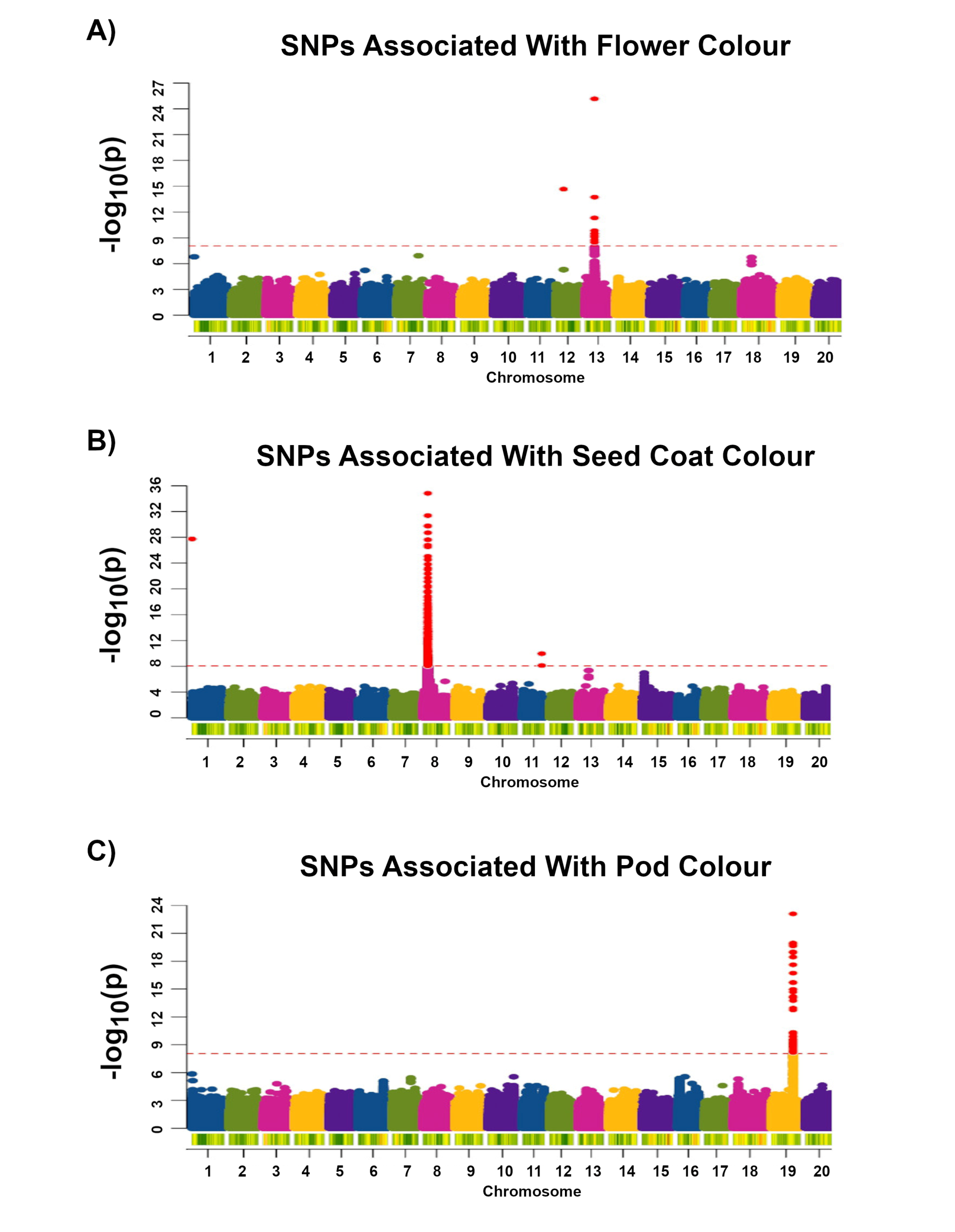
**Supplementary Figure 1.** P-value of each SNPs association for **a)** flower colour **b)** seed coat colour **c)** pod colour in the soybean VCF. SNPs coloured red have been determined as significantly associated for the given trait as they have a p-value less than the -log10(8) significance threshold for this GWAS.


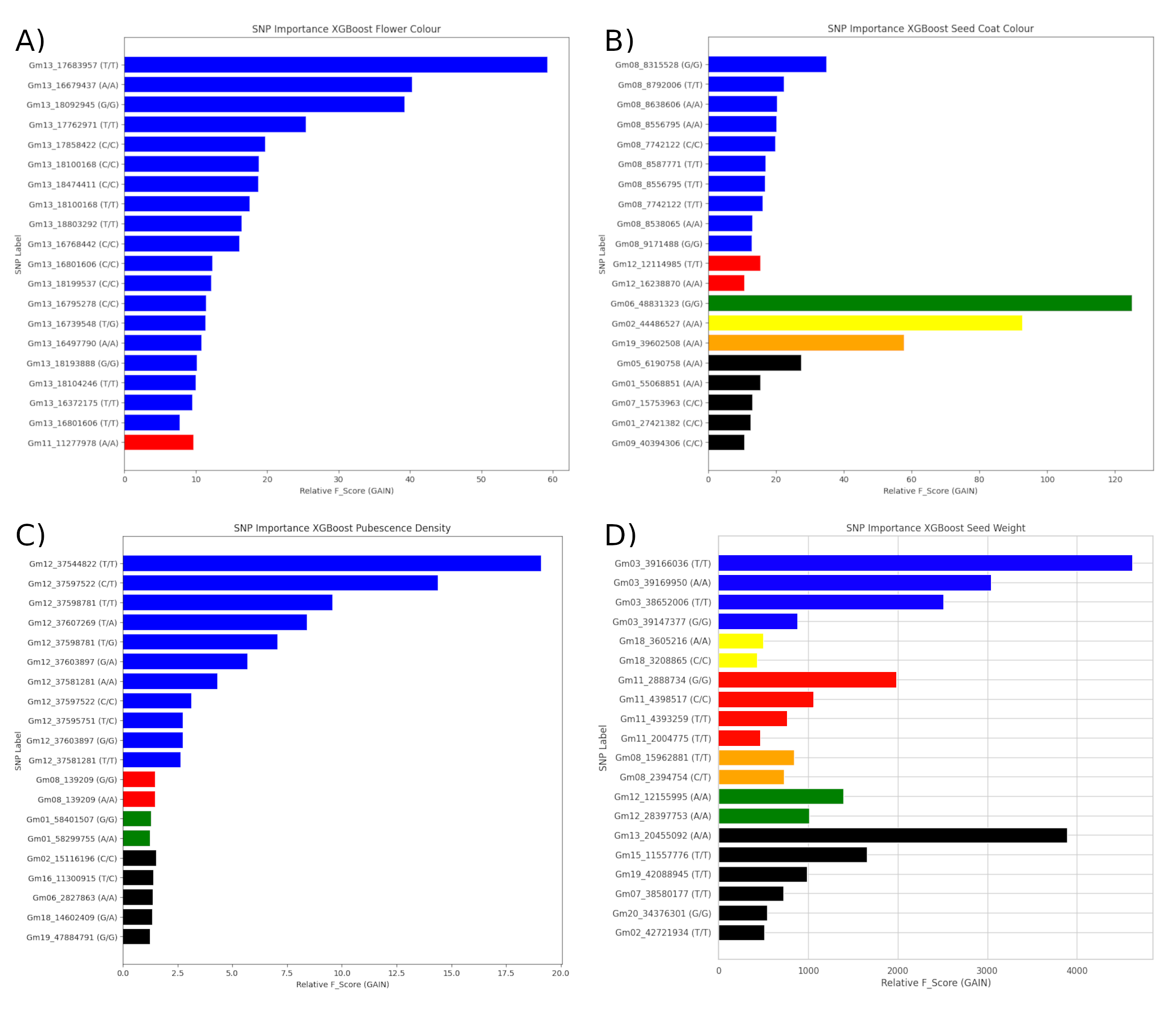
**Supplementary Figure 2.** Graphs ranking the top 20 most input SNPs by gain as identified by XGBoost models for trait predictions for traits with regions of importance identified from XGBoost. Blue bars are region of importance, whereas other colours represent collections of important SNPs on the same chromosome. Black bars represent left over SNPs with no relation to other SNPs in the ranking. SNP rankings for genome wide SNP input for **A)** flower colour **B)** seed coat colour **C)** pubescence density **D)** seed weight.


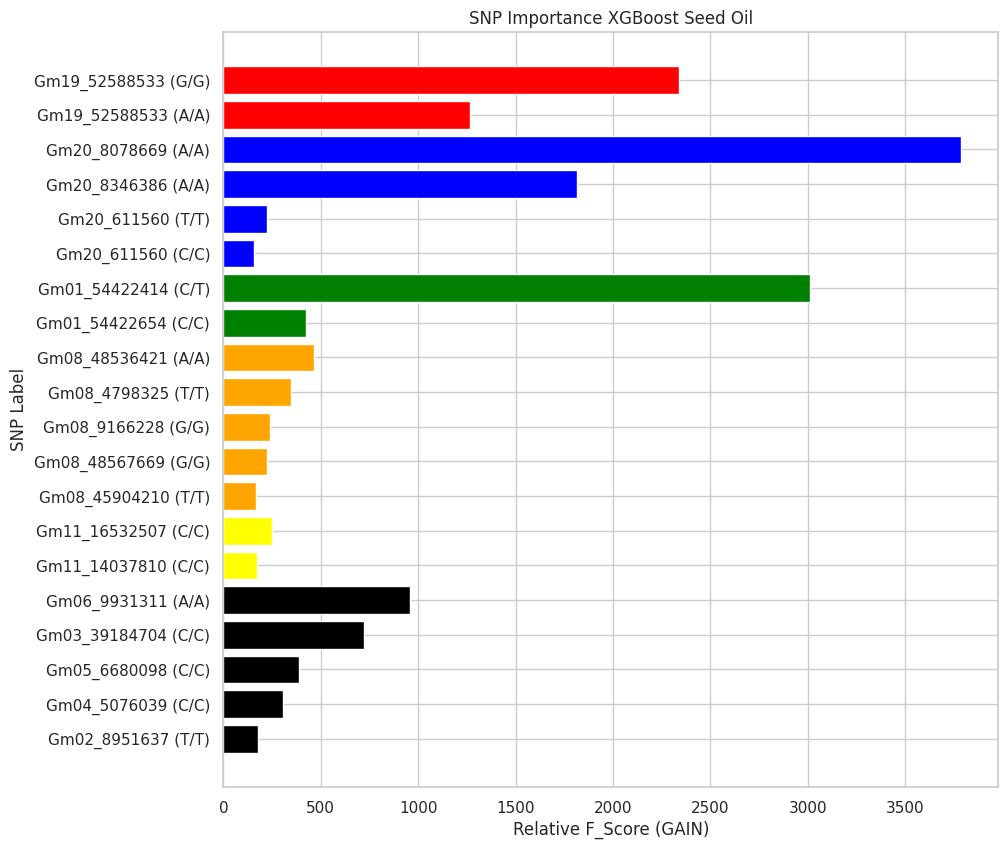


**Supp Figure 3.** Top 20 ranked SNPs for XGBoost Seed Oil Prediction


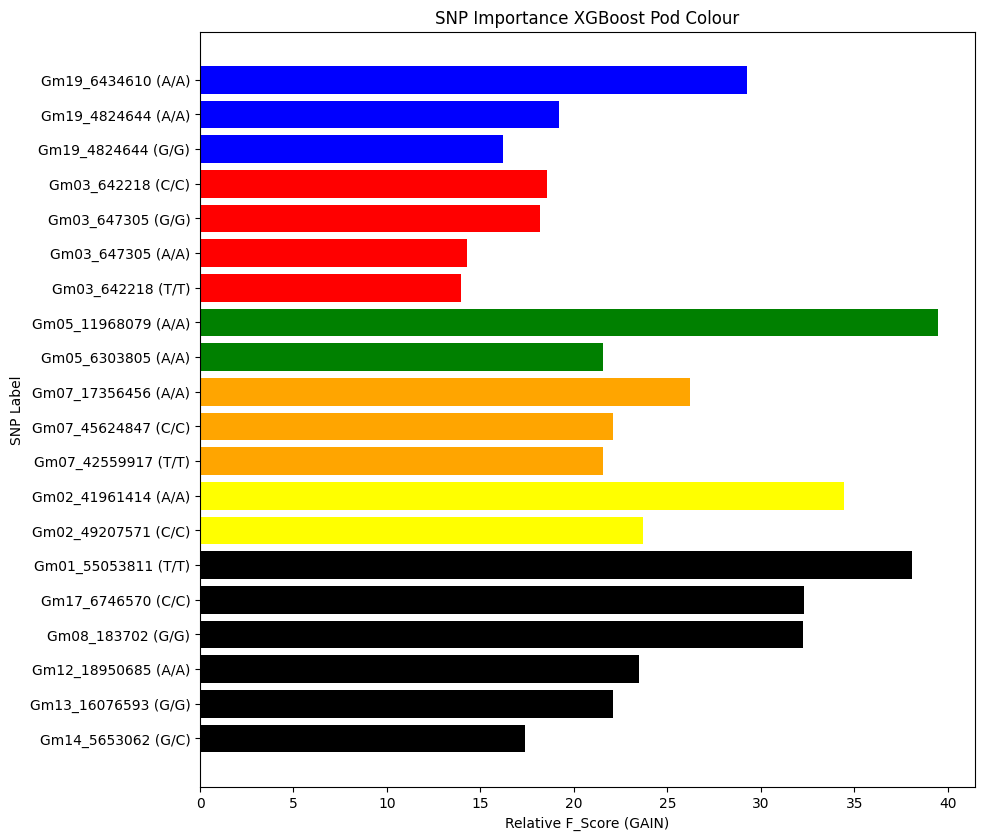


**Supp Figure 4.** Top 20 ranked SNPs for XGBoost Pod Colour Prediction


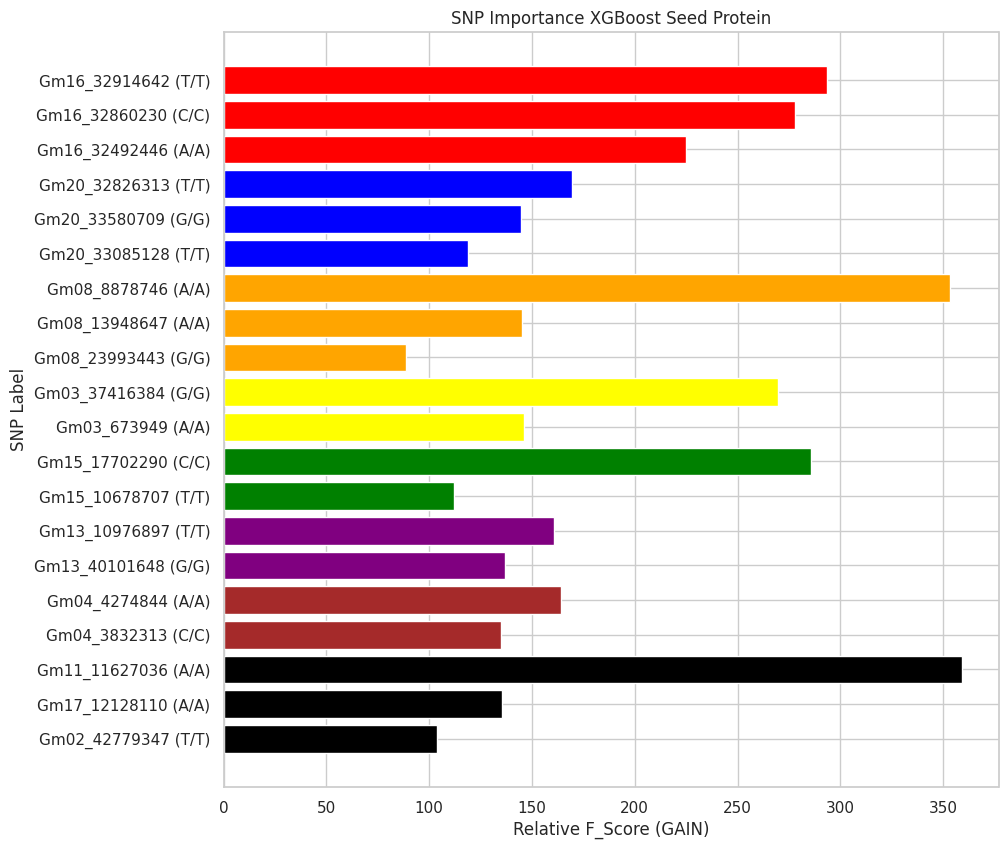


**Supp Figure 5.** Top 20 ranked SNPs for XGBoost Seed Protein Prediction
